# Signer-Independent Arabic Sign Language Recognition System Using Deep Learning Model

**DOI:** 10.3390/s23167156

**Published:** 2023-08-14

**Authors:** Kanchon Kanti Podder, Maymouna Ezeddin, Muhammad E. H. Chowdhury, Md. Shaheenur Islam Sumon, Anas M. Tahir, Mohamed Arselene Ayari, Proma Dutta, Amith Khandakar, Zaid Bin Mahbub, Muhammad Abdul Kadir

**Affiliations:** 1 Department of Biomedical Physics & Technology, University of Dhaka, Dhaka 1000, Bangladesh; 2 Department of Computer Science, Hamad Bin Khalifa University, Doha 34110, Qatar; 3 Department of Electrical Engineering, Qatar University, Doha 2713, Qatar; 4 Department of Biomedical Engineering, Military Institute of Science and Technology (MIST), Dhaka 1216, Bangladesh; 5 Department of Civil and Architectural Engineering, Qatar University, Doha 2713, Qatar; 6 Department of Electrical& Electronic Engineering, Chittagong University of Engineering & Technology, Chittagong 4349, Bangladesh; 7 Department of Mathematics and Physics, North South University, Dhaka 1229, Bangladesh

**Keywords:** Arabic Sign Language, deep learning, dynamic sign language, segmentation, MediaPipe

## Abstract

Every one of us has a unique manner of communicating to explore the world, and such communication helps to interpret life. Sign language is the popular language of communication for hearing and speech-disabled people. When a sign language user interacts with a non-sign language user, it becomes difficult for a signer to express themselves to another person. A sign language recognition system can help a signer to interpret the sign of a non-sign language user. This study presents a sign language recognition system that is capable of recognizing Arabic Sign Language from recorded RGB videos. To achieve this, two datasets were considered, such as (1) the raw dataset and (2) the face–hand region-based segmented dataset produced from the raw dataset. Moreover, operational layer-based multi-layer perceptron “SelfMLP” is proposed in this study to build CNN-LSTM-SelfMLP models for Arabic Sign Language recognition. MobileNetV2 and ResNet18-based CNN backbones and three SelfMLPs were used to construct six different models of CNN-LSTM-SelfMLP architecture for performance comparison of Arabic Sign Language recognition. This study examined the signer-independent mode to deal with real-time application circumstances. As a result, MobileNetV2-LSTM-SelfMLP on the segmented dataset achieved the best accuracy of 87.69% with 88.57% precision, 87.69% recall, 87.72% F1 score, and 99.75% specificity. Overall, face–hand region-based segmentation and SelfMLP-infused MobileNetV2-LSTM-SelfMLP surpassed the previous findings on Arabic Sign Language recognition by 10.970% accuracy.

## 1. Introduction

Sign language serves as a vital means of communication for individuals who are deaf or hard of hearing. As of 2018, the number of people with deaf and hard-hearing disorders reached 470 million, comprising approximately 6.1 percent of the global population. This number is projected to surpass 900 million by 2050 [1]. Sign language is a distinct language that enables communication through visual gestures and body movements. It is important to note that sign language is not equivalent to body language or generic gestures; it is a full-fledged linguistic system in its own right.

Lack of awareness and understanding of sign language creates significant barriers between deaf individuals and society. Unlike spoken languages, sign languages do not have a direct link to any particular spoken language. Moreover, different sign languages, such as American Sign Language (ASL) and British Sign Language (BSL), are not mere dialects but distinct languages [2]. Furthermore, within a single country, multiple sign languages can exist, analogous to the presence of various spoken language dialects [3].

Recent advancements in the fields of computer vision and deep learning have contributed to the development of several digital assistance systems. These systems have facilitated diverse applications, including leaf disease detection [4] and, notably, sign language recognition [5,6,7], synthesis, and translation [5,6,7,8]. These technological breakthroughs have greatly enhanced communication between deaf individuals and society. Significant progress has been made in the development of state-of-the-art models for sign language recognition systems. Computer vision techniques, coupled with deep learning approaches, have enabled effective recognition of static and dynamic signs in sign language. Static signs are characterized by the shape and orientation of the signer’s hands and fingers, without involving motion [5,7,9]. These signs typically represent finger spelling of alphabet letters and numerals in most sign languages. Recognition of static sign language involves the use of advanced CNN models, such as MobileNetV2, ResNet18, and EfficientNet_B1, while hand segmentation techniques can employ DenseNet201 FPN, M-UNET, or UNET [5,7].

In contrast, dynamic signs encompass the motion of the hands and other body parts during signing [10]. Dynamic sign language recognition relies on the extraction and analysis of spatio-temporal data, which captures the sequential and temporal aspects of signing. CNN-LSTM architectures have proven effective in this regard, as CNNs can extract spatial features from video frames, which are subsequently fed into LSTM models for temporal feature extraction [11].

Sign gestures are mostly dynamic, where movement plays a crucial role in conveying the meaning. Dynamic sign language can be manual, non-manual, or a combination of the two. Hand and body motion gestures are examples of manual gestures. On the contrary, non-manual gestures rely on other aspects of the body, such as facial expressions and head movement, to express thoughts and clarify or accentuate meaning [12]. The majority of signs use both manual and non-manual movements.

Facial expressions are the most prominent component of non-manual sign language that poses several key advantages. First, facial expressions rely on mouths, eyes, brows, lips, noses, and cheeks to portray feelings and emotions that motor movements cannot convey. Moreover, they are crucial in expressing the grammatical peculiarities of sign languages. Furthermore, they are employed for grammatical structure, lexical distinction, and discourse functions such as negation, as well as adverbial and adjectival content [13]. Non-manual movements frequently employ lip patterns as a criterion. However, only a few lip patterns are specific to sign languages, whereas most lip patterns correspond to the spoken language pronunciation of signed words. Deaf people are excellent lip readers, reading lip patterns to fully comprehend the signs, especially from hearing persons. Other key components of facial expressions are the brows and forehead. They can be used on their own or in conjunction with other facial expression elements such as lip patterns. Moreover, the non-manual articulators of sign languages rely heavily on head movements. Head posture can be used alone or in combination with manual motions in Arabic Sign Language, which combines hand gestures and head motion.

The deaf and mute community in Arab countries uses Arabic Sign Language (ArSL). This language is a synthesis of numerous sign languages used in Arabic-speaking nations [14]. The League of Arab States (LAS) and the Arab League Educational, Cultural, and Scientific Organization (ALECSO) introduced it in 1999, and a 3200-sign dictionary was published in two parts in 2000 and 2006 [15]. This language is primarily spoken in Arab Gulf countries and is the primary language of media outlets such as Al-Jazeera.

The absence of databases with sufficient numbers of relevant videos reflecting diverse articulators of the sign language [14] is one of the key issues connected with ArSL recognition systems. Therefore, a multi-modality ArSL database was proposed in [16], with a focus on manual and non-manual gestures. For real-life implementation, the ArSL recognition system on RGB videos captured from a webcam, a smartphone, and other devices can be useful. So, only the RGB videos from the dataset of [16] are used in this study. By addressing the issues related to real-time ArSL recognition, the contribution of this study is as follows:

- An easy and faster face and hand region segmentation method using MediaPipe Holistic is proposed in this study. A segmented dataset is created using this method for classification purposes.
- An LSTM module of two layers and a smaller hidden size is proposed in this study to extract the temporal information embedded in face and hand movement.
- A modified multi-layer perceptron (MLP) using operational layers, the SelfMLP classifier, is proposed in this study for the classification task. Two-layer SelfMLP with fewer parameters can address the over-fitting problem.
- A novel CNN-LSTM-SelfMLP architecture is proposed in this study, where state-of-the-art MobileNetV2 and ResNet18 are used as the convolutional neural network (CNN) backbone.
- The proposed models are lightweight with an optimum number of parameters and less time-consuming in inference tasks.
- The proposed model MobileNetV2-LSTM-SelfMLP outperformed the previous literature by a significant margin.

The rest of the paper is organized as follows: In Section 2 the relevant works related to non-manual features are reviewed. Section 3 presents the adopted methodology and the used materials. Section 4 presents the results and discusses the findings. Lastly, the conclusions are drawn in Section 5.

## 2. Literature Review

In the last two decades, numerous systems for the automatic recognition of sign language have been proposed [5,6,7,17]. The majority of these strategies focused on the prominent aspects of physical motions. On the contrary, few studies have investigated non-manual variables (such as facial expressions) alone or combined with manual features [16]. The paucity of datasets, especially for ArSL, is one of the main limitations of recognition systems. This section illustrates the publicly available Arabic Sign Language datasets, as well as the most relevant sign language recognition techniques.

A key challenge to enhance the current sign language recognition and translation systems is the lack of databases [8]. The three main types of sign language databases are finger spelling, isolated signs, and continuous signs. Most digits and alphabets in sign languages are static and can only be represented with fingers. An image-based manual alphabet in ArSL recognition was used in [18], where feature vector extraction and adaptive neuro fuzzy inference system (ANFIS) were used to gain an accuracy of 93.55%. A similar ArSL alphabet recognition is reported in [19] by claiming better performance using polynomial classifiers than an ANFIS-based ArSL recognition system on the same dataset. But, the training data used in the study [19] was not uniform, and that makes the probability of designing a better sign language recognition system using a new and versatile dataset.

In Reference [20], researchers extended the ArSL alphabet recognition by introducing voice translation of ArSL recognition for bridging sign language with non-sign language users. Though the prewitt edge identifier and principal component analysis (PCA) algorithm were used with an accuracy of 97% in the study [20] for recognition, misrecognition was reported whenever high or poor illumination occurred. Further in [21], hand movement of a gesture was tracked based on the reference of the detected face with more than 95% accuracy using the hidden Markov model (HMM). Additionally, one-versus-all SVM utilizing a histogram of oriented gradients (HOG) descriptors was also adopted for static Arabic sign alphabet recognition. But, the static Arabic sign alphabet recognition creates a research gap that addresses the limitation of the use of the sign alphabet in a real-life scenario. Almost all the signs used in real-life conversations are dynamic in nature. These proposed models have limitations in training data uniformity, susceptibility to lighting conditions, or misrecognition of dynamic signs in real-life scenarios. Computer vision has become increasingly prevalent in recent years, with applications in a wide range of domains, including sign language recognition and sports analytics. One such application is the recognition of basketball referee signals from real-time videos. This computer vision problem involves developing methodologies that can effectively identify and interpret the hand signals performed by basketball referees during live games without limitations of lighting conditions and misrecognition of hand gestures.

In [22], the researchers proposed a method for recognizing basketball referee hand signals from recorded game recordings using image segmentation based on the histogram of oriented gradients (HOG) and local binary pattern (LBP) features. Using LBP features and a support vector machine (SVM) for classification, the proposed method obtained a 95.6% accuracy rate. In [23], the researchers employed the Leap Motion device to monitor the hand and finger movements of the user and the hidden Markov classification (HMC) algorithm to identify the corresponding gestures. Utilizing data from the Leap Motion device and the HMC algorithm, the system was capable of recognizing gestures. This paper described a subsystem for gesture recognition that receives data from the Leap Motion device and returns results to the user. There are three states in the system: motion detection, gesture recognition, and data purging. The article employs words per minute (WPM) as a performance metric and minimum string distance (MSD) as an error rate.

Isolated sign words can be static or dynamic, and they have the same meaning as spoken words. In continuous sign language databases, more than one sign word is performed constantly. The vertical movement of the head and hands is correlated in manual and non-manual sign language recognition [24]. Similar manual but different non-manual sign video recognition is investigated in the study in [25]. This study led to the conclusion of achieving an accuracy of 73% for 20 classes (20 signs) and 80.25% for 2 classes (2 signs). Combining features of facial expression with gestures on sign language recognition exhibits a boost in the accuracy [26]. The use of CNN followed by long short-term memory (LSTM) or bidirectional LSTM (BiLSTM) is one of the popular techniques in sign language recognition [11,27,28,29]. In [29], an Arabic Sign Language recognition system was proposed with attention-based CNN-BiLSTM, which was capable of recognizing dynamic sign videos and static sign images with 85.60% accuracy in signer-independent mode. This system achieved high accuracy on the dataset [30], which was versatile in context as variable lighting, different clothing, and different distances from the camera were maintained [29], but the number of classes for dynamic sign investigation was 40. When analyzing a sign language database, the variability (signer’s number), number of samples per sign (size), and sign representation of the database must all be taken into account. Another study investigating 80 static and dynamic sign videos from 40 signers using 2D body and hand skeleton data [31] achieved 88.09% accuracy in signer-independent mode.

The two-dimensional (2D) convolutional recurrent neural network (2D-CRNN) and the 3D convolutional neural network (3D-CNN) were also adopted by researchers to achieve 92% and 99% accuracy, but the dataset consisted of 224 videos of five signers executing 56 distinct signs [32]. Three-dimensional (3D) GS-NET is another research in Arabic Sign Language recognition, where the system can recognize signs from RGB videos [33]. The authors of Reference [34] also introduced a dataset called ArabSign. The researchers gathered a dataset consisting of 9335 video samples from six individuals. They proceeded to train an encoder–decoder model to accurately identify sentences from sign language videos. The model achieved an average word error rate (WER) of 0.50. In Reference [35], a study by the author introduced a pose-based Transformer model for the purpose of recognizing a more extensive dataset called KArSL-100. This dataset encompassed 100 distinct classes and was specifically designed for the task of sign video recognition. The researchers in Reference [35] attained an accuracy rate of 68.2% in signer-independent mode. Similarly, the mArSL dataset of 6748 videos of 50 classes performed by 4 signers was proposed in [16] which is a multi-modality dataset containing RGB videos, depth data, skeleton joint points, and face information of manual and non-manual signs. A fusion-based technique of CNN-LSTM for optical flow and LSTM for animation units was used in that study, which achieved 76% of accuracy, but showed an accuracy of only 58.9% for the first signer in signer-independent mode [16]. The aforementioned works require multiple pre-processing, complex architecture and Kinect sensors. Even though some models show reasonable performance on a small dataset, such a complex approach can have limitations in real-life implementations because of using heavy networks and sophisticated sensors.

## 3. Methods and Materials

**Figure 1:**
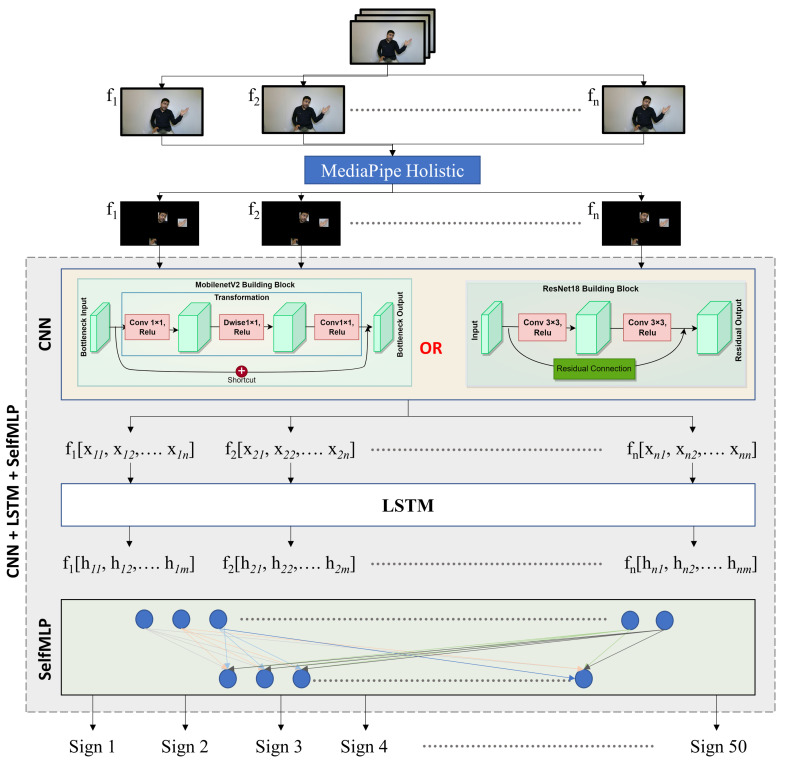
System architecture for segmented Arabic sign recognition system.

The proposed Arabic Sign Language recognition systems were tested in a signer-independent setting. Thus, the model was evaluated on how well it performs on an unseen test set that it has not been exposed to during the training phase. This kind of setting mimics real-life applications where the model needs to reliably recognize signs on unseen signers. This research was focused on face–hand segmented video data for Arabic sign recognition. To further validate the importance of region of interest (ROI) segmentation, all models were tested on both segmented and unsegmented raw videos. Figure 1 illustrates the proposed pipeline for segmented sign video data recognition.

### 3.1. Dataset Description

The dataset used in this study combines manual and non-manual gestures for signs [16]. Inter and intra-class similarities are involved in this dataset. To maintain real-world conditions, the data were recorded in a control-free environment with variations in subjects’ clothing color over multiple sessions.

The Kinect V2 acquisition system (motion sensing device by Microsoft) [36] was used, where an infra-red (IR) camera and an IR projector were used as the depth sensor along with a color camera at a distance of 1.5 m from the signer. The recorded signs consist of the signer’s face and the upper portion of the body. In this study, only the color video data were used. Each frame of the videos was considered in RGB and avi format.

**Figure 2:**
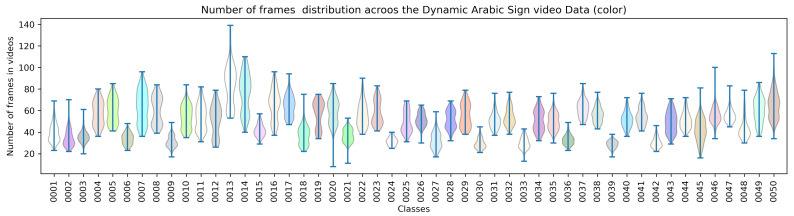
A graphical description of number of frames distribution in inter and intra-class Arabic sign color video data in [16].

The sign videos for a particular sign contain different numbers of frames. Figure 2 graphically describes the total number of frames distributed in each video. The variable number of frames per recorded video is shown, as the sign performance time varies between subjects, and it even slightly varies for different sessions of the same subject. As illustrated in Figure 2, Classes 0013, 0014, 0020, 0045, 0047, and 0050 contain a wide range of total number of frames in each video, while Classes 0024, 0030, and 0039 contain a close distribution of the number of frames in each class for all four signers in different settings of recording.

### 3.2. Pre-Processing

In this study, the dataset was pre-processed by taking the face and hand region as ROI. MediaPipe Holistic was used for ROI selection [37]. All the video frames were resized to 224 × 224 RGB. Moreover, each video was pre-processed to a fixed number of frames.

#### 3.2.1. MediaPipe Holistic

**Figure 3:**
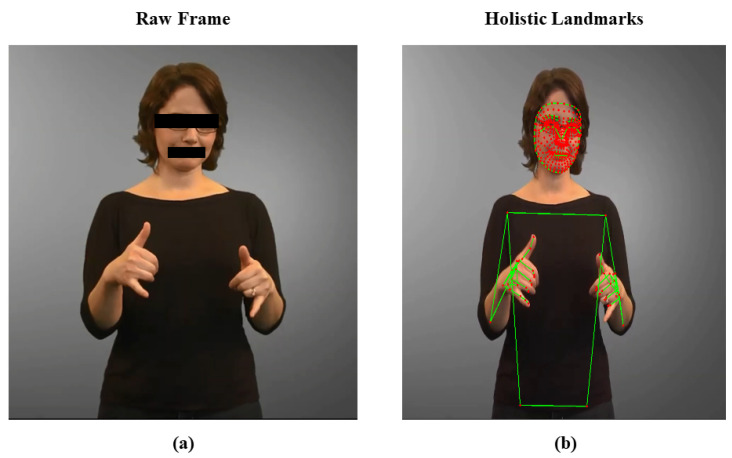
Detecting landmarks and connections of face, hand, and upper body pose on human subjects: (**a**) Raw frame; (**b**) Frame with landmarks and connections.

MediaPipe [37], a library created by Google called Holistic, includes various pose, hand, and face landmark detection models. For all three components, it offers over 540 landmarks with X, Y, and Z coordinates. While the Z coordinate denotes the distance of the landmark from the camera, the X and Y coordinates indicate the location of the landmarks in the horizontal and vertical planes, respectively. Using a two-stage pipeline, the landmarks are retrieved from video frames. To identify the main body joints, a coarse pose estimation is performed in the initial stage. The second stage involves finding the fine-grained landmarks around the joints. The position, hands, and face of the individual are then tracked in real time using the landmarks. An effective tool for tracking and estimating human stance is MediaPipe Holistic. It can be applied to many different things, including virtual reality, sports analytics, and gesture detection. Figure 3 shows an upper body pose, face, and hand landmark detection using MediaPipe Holistic on a human subject performing American Sign Language (ASL) in a video collected from [38].

#### 3.2.2. Hand-Face Region Segmented Video Data

**Figure 4:**
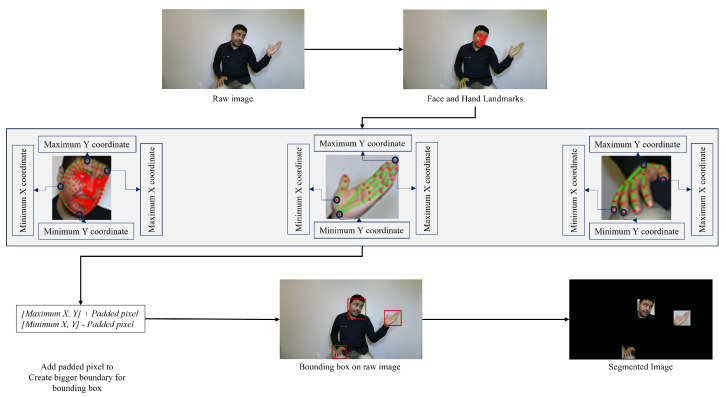
Segmented frame/image data creation using MediaPipe Holistic module.

In this study, we segmented the face and hand regions of signers to remove the backgrounds. Figure 4 illustrates the proposed video segmentation pipeline. The segmented frame video was processed in a step-by-step manner. First, all the landmarks for the face, right hand, and left hand were tracked separately. Then, bounding boxes were created for the face, right hand, and left hand. To accomplish this, three sets of [(maximumx,y),(minimumx,y)] coordinates were found for all the face, right-hand, and left-hand landmarks. These coordinates were used to generate the bounding boxes for the face, right hand, and left hand. However, these bounding boxes had hard edges, which did not capture the full region of interest. To address this, the bounding boxes were slightly enlarged to capture the full region of interest. This was accomplished by adding padded pixels=20 to the (maximumx,y) coordinates and subtracting paddedpixels=20 from the (minimumx,y) coordinates. This resulted in three new bounding boxes, which were then created on the raw image. The regions of interest were then cropped out from these bounding boxes, as shown in Figure 4. Frame-by-frame segmented frames/images were concatenated to create the segmented video.

#### 3.2.3. Fixing the Number of Frames in Each Video

**Figure 5:**
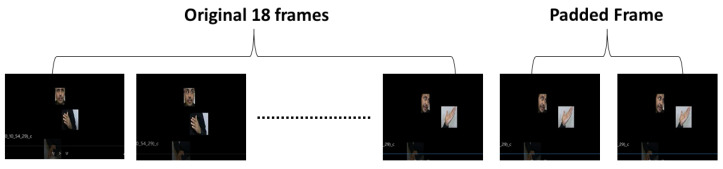
Pre-processing of sign video data to upsampled to 20 frames by adding last frame.

For Arabic sign recognition, a fixed number of frames per video was used as input to the deep learning model. All video data were pre-processed to 20 frames. Among 6667 videos available in the dataset [16], 14 videos that had less than 20 frames were pre-processed by adding the last N frame(s) until it matched 20 frames. Figure 5 illustrates the process of video upsampling using padded frames. In total, 6651 videos had more than 20 frames, and these were downsampled to 20 frames. A selection of these frames was made by calculating the interval between subsequent frames in relation to the total number of sign frames in the sequence.

### 3.3. Classification Model Selection

Spatial and temporal features are the two components of dynamic sign videos. The hand shape and the facial expression of signers in each frame of the sign video are the spatial features, while the movement of the hand shapes and changes in facial expression over time are the temporal features of Arabic Sign Language recognition. Convolutional and LSTM layers were used for spatial and temporal feature extraction, respectively. Moreover, self-MLP layers were used for the final classification. The details of CNN, LSTM, and self-MLP are given below:

#### 3.3.1. Convolutional Neural Networks (CNNs)

**Figure 6:**
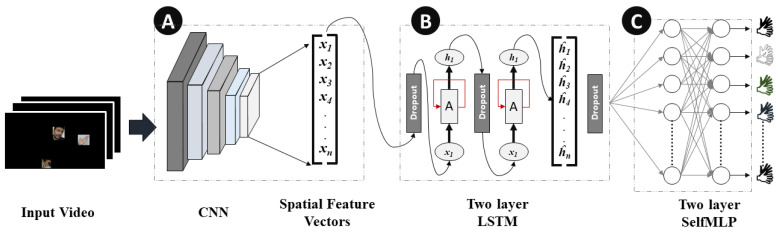
CNN-LSTM-SelfMLP-based Arabic Sign Language recognition system architecture. Here (**A**) Spatial feature extractor, (**B**) Two Layer LSTM and (**C**) Two layer SelfMLP.

MobileNetV2 [39] and ResNet18 [40] are among the top CNN models that showed state-of-the-art performance on ImageNet challenge [41]. In this research, both of the pre-trained models were fine-tuned on the mArSL dataset, which were previously trained on 21,841 subcategories. The last classification MLP layer was removed, and the spatial features were flattened to be fed to LSTM layers. In Figure 6, Block A represents the spatial feature extraction by CNN models in the CNN-LSTM-SelfMLP-based system architecture.

#### 3.3.2. Long Short-Term Memory (LSTM)

In dynamic sign videos, there is a degree of temporal connection between consecutive frames. Neural networks such as vanilla recurrent neural network (RNN), LSTM, and gated recurrent unit (GRU), are capable of finding the temporal connections in video data. LSTM [42] layers are widely used in different sign language recognition [27,28,43]. LSTM performs better than vanilla RNN in this task, where information on the previous frames can be preserved by understanding the present frame. The LSTM layer has cell states and hidden states, which enables LSTM to add or remove information by regulating gates using cell states. Moreover, LSTM resolves the vanishing gradient problem of vanilla RNN by possessing the additive gradient mechanism [42]. Two layers of LSTM with 310 hidden states with a 30% dropout rate were used in this study for temporal feature extraction. Figure 6 represents the two-layer-based LSTM module in the CNN-LSTM-SelfMLP-based system architecture.

#### 3.3.3. Self-MLP

Operational neural networks (ONNs) and the new variant of self-organized operational neural networks (self-ONNs) have recently been proposed to address the well-known limitations and drawbacks of conventional convolutional neural networks (CNNs), such as network homogeneity with the sole linear neuron model. Self-ONN achieves an ultimate heterogeneity level to boost the network diversity while maintaining computational efficiency. Self-ONN with generative neurons can adapt (optimize) the nodal operator of each connection during the training process. Any non-linear function can be approximated near a point with qth-order Taylor approximation.

(1) $$f\left(x\right)=f\left(x_{0}\right)+\frac{f^{\prime }\left(x_{0}\right)}{1!}\left(x-x_{0}\right)+\frac{f^{\prime \prime }\left(x_{0}\right)}{2!}{\left(x-x_{0}\right)}^{2}+....+\frac{f^{q}\left(x_{0}\right)}{q!}{\left(x-x_{0}\right)}^{q}$$

Equation (1) can be bounded to [−1,1] by tanh or [0,1] by sinh and approximated to 0. The equation after approximation can be expressed as,

(2) $$f\left(x\right)=f\left(0\right)+\frac{f^{\prime }\left(x_{0}\right)}{1!}\left(x\right)+\frac{f^{\prime \prime }\left(x_{0}\right)}{2!}{\left(x\right)}^{2}+....+\frac{f^{q}\left(x_{0}\right)}{q!}{\left(x\right)}^{q}$$

(3) $$f\left(x\right)=b+w_{1}\left(x\right)+w_{2}{\left(x\right)}^{2}+....+w_{q}{\left(x\right)}^{q}$$

The $w_{1},w_{2},...,w_{q}$ coefficient values in Equation (3) are optimized in the back-propagation process. Any ONN operation can be expressed as [44],

(4) $$\hat{x_{r}^{k}}\left(i,j\right)=P_{n}^{k}(\psi_{r}^{k}{\left(w_{r}^{k}\left(i,j\right),y_{r-1}\left(i-u,j-v\right)\right)}_{\left(u,v)=(0,0\right)}^{\left(p-1,q-1\right)}$$

**Figure 7:**
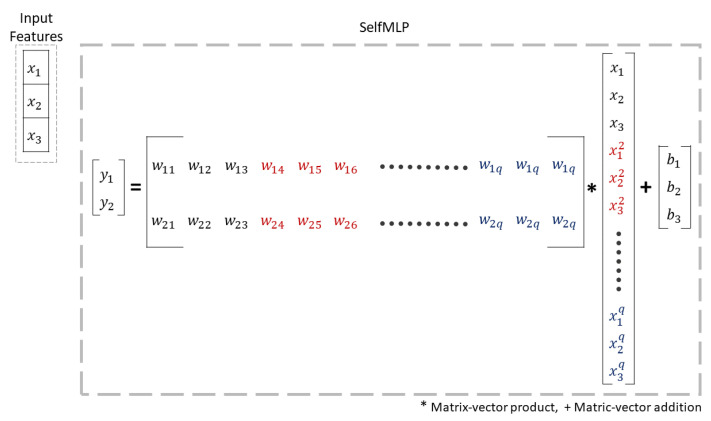
The illustration of q order ONN operation of weight, input features, and biases.

Here, in Equation (4), $P_{r}^{k}\left(\;\cdot \;\right):{\;R}^{MN\times k^{2}}\to {\;R}^{MN}$ is the pool operator and $\psi_{r}^{k}\left(\;\cdot \;\right):{\;R}^{MN\times k^{2}}\to {\;R}^{MN\times k^{2}}$ is the nodal operator. The drawback of choosing a proper nodal operator in the original ONN paper is addressed in self-ONN and solved by iteratively generated nodal operators. So, if $x_{1},x_{2},$ and $x_{3}$ are the input features, then self-ONN will create *q* order copies of inputs and also generate *q* filter-banks, as shown in Figure 7. A more theoretical explanation of ONN and self-ONN can be found in References [44,45], respectively.

**Figure 8:**
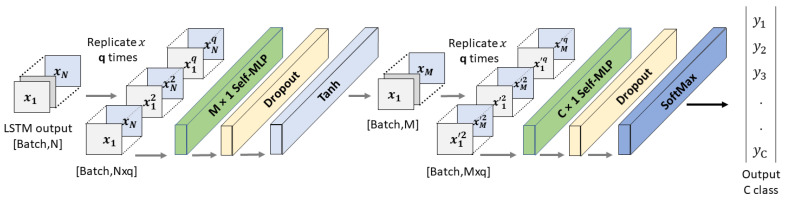
Architecture of self-MLP classifier.

Figure 8 illustrates the operations of self-MLP layers that exist in Figure 6 Block B. MLP layers can be implemented using convolutional layers by using kernels with the exact same size as the input. Thus, a single sliding window of the convolutional kernel will cover the full signal, retaining the fully connected nature of MLPs. In a similar manner, 1D Self ONN layers can be used to implement self-MLP layers. As K×1 features are propagating from LSTM to self-MLP, K×1 kernels were used to mimic the self-MLP neurons. Moreover, dropouts were used for countering the over-fitting problem, while tanh was used as the activation function.

### 3.4. Experimental Setup

A good method for evaluating the model on various dataset chunks is cross-validation. Based on the signer-independent mode, the four-fold cross-validation technique was used in this investigation. The data from one signer’s video was thus kept for the test set, while the remaining videos from the other three signers were kept for training and validation. The major goal of such a study is to assess the model using entirely new data, then simulate and test them in real-world situations. We used all the videos from the three signers to create a training and validation set, and then we split that set in two: 90% of the videos from the three signers went into the training set, while the other 10% went into the validation set.

The CNN model was unfrozen, so all of its parameters were fine-tuned during the training process. The CNN model was initialized with ImageNet parameters at the start of the training process, while the parameters of the LSTM-SelfMLP part of CNN-LSTM-SelfMLP were randomly initialized. After the first iteration of the first epoch, all of the parameters in the CNN-LSTM-SelfMLP architecture were fine-tuned. Fine-tuning is a technique that is used to improve the performance of a pre-trained model on a new task. In this case, the CNN model was pre-trained on the ImageNet dataset, which contains images of objects from a variety of categories. The LSTM-SelfMLP part of CNN-LSTM-SelfMLP was randomly initialized, which means that they were not trained on any data before the fine-tuning process.

This study was carried out with the Pytorch package and Python 3.7. Google ColabPro was used to train all of the models, and the specifications of ColabPro were 16 GB Tesla T4 GPU and 120 GB high RAM. Table 1 shows the training settings that were employed in this experiment.

### 3.5. Evaluation Metrics for Arabic Sign Language Recognition

The performance of the Arabic Sign Language recognition system is assessed using five evaluation metrics, namely, overall accuracy, precision, sensitivity, F1-score, and specificity. Moreover, computational complexity analysis is performed by comparing the models in terms of inference time and the number of trainable parameters.

For all metrics formulations, TP = number of true-positive instances, TN = number of true-negative instances, FN = number of false-negative instances, and FP = number of false-positive instances. Moreover, we can define, α = TP+TN+FP+FN, β = TP+FP, θ = TP+FN, and γ = TN+FP.

Accordingly, the overall accuracy presents the ratio of the correctly classified videos among the entire video dataset.

(5) $$Overall Accuracy=\frac{TP}{\alpha }$$

The precision is the rate of correctly classified positive class videos samples among all the videos classified as positive samples.

(6) $$\mathrm{Precision}=\frac{TP}{\beta }$$

The sensitivity is the rate of correctly predicted positive samples from among the positive class samples.

(7) $$\mathrm{Recall}=\frac{TP}{\theta }$$

A model can perform good at precise recognition, but poor sensitivity and vice-versa. For harmonic mean of precision and recall of any model, F1 score is calculated as given in Equation (8). F1 score represents the ratio of multiples of precision and sensitivity to the summation of the same parameters.

(8) $$F1\;\mathrm{Score}=\frac{2\times (Precision\times Recall)}{Precision+Recall}$$

Ultimately, specificity is the indicator of model performance in the meaning of model true-negative classification over the true-negative and false-positive combination. Specificity can be represented as given in Equation (9).

(9) $$\mathrm{Specificity}=\frac{TN}{\gamma }$$

The ROC curve is the probability curve that plots the true-positive rate against the false-positive rate at various threshold values of a model’s prediction. The “One vs. Rest” method is suitable for ROC curve plotting for multi-class problems, and the average of all the “One vs. Rest” plots represents the overall model performance of the true-positive prediction rate against the false-positive prediction rate. The area covered under the curve, or the AUC, is used to summarize the ROC curve by implicating the ability of a classifier in distinguishing between classes.

The utilization of a confidence interval allows for the quantification of the level of uncertainty pertaining to the accuracy of the model. For instance, when a confidence interval of 95% is established, it indicates that there is a 95% level of confidence that the actual accuracy of the model falls within the bounds of the confidence interval. The z-score is a statistical measure that quantifies the deviation of a specific value from the mean. In this particular instance, the z-score is employed to determine the appropriate confidence level. For instance, when aiming to establish a confidence interval with a confidence level of 95%, a z-score of 1.96 would be employed. The standard error of the estimate is a statistical metric that quantifies the extent of variability in the precision of the model. The calculation involves the extraction of the square root of the variance of the accuracy. The sample size refers to the quantity of videos employed for training the model. As the number of samples increases, the width of the confidence interval decreases. The equation of the standard error is given below:

(10) $$SE=\sqrt{\frac{p*(1-p)}{n}}$$

where SE is the standard error, *p* is the accuracy of the model, and *n* is the total number of samples. If, $z_{score}$ is the z-score for the M% confidence interval, then the confidence interval CI will be,

(11) $$CI=p\pm z_{score}*SE$$

## 4. Results and Discussion

In this study, six models were trained with two different spatial feature extractors and three different self-MLP classifiers. The classifier with q = 1, conventional MLP, has around 4.98 million trainable parameters, while the model complexity slightly increases with additional 23 thousand and 46 thousand parameters for q = 3 and q = 5 self-MLP classifiers, respectively. The number of layers and neurons was fixed for all three *q* values. ResNet18-LSTM-SelfMLP (q = 3) is the most complex model and MobileNetV2-LSTM-SelfMLP (q = 1) is the lightest model. Table 2 presents the model complexity in terms of trainable parameters for all six combinations for both raw and segmented dataset. The accuracy and loss curves for all twelve models can be found in Supplementary Tables S1–S12. By comparing Supplementary Tables S1–S6 with Supplementary Tables S7–S12, it is evident that the validation accuracy and loss curves for all six models on the segmented dataset followed the training curves better than the models with same combinations used on the raw dataset.

**Figure 9:**
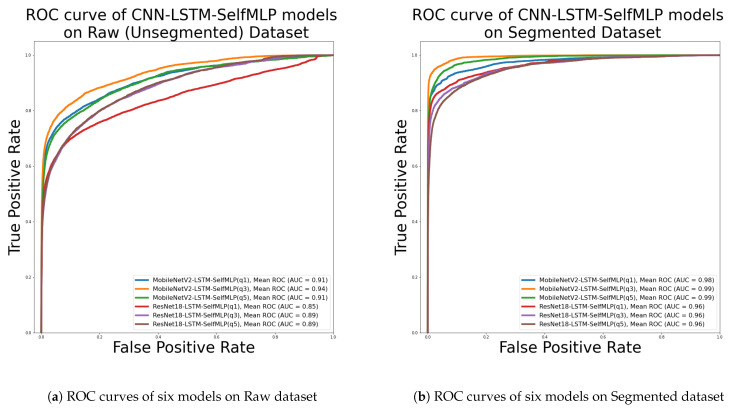
Receiver operating characteristic (ROC) curves of six CNN-LSTM-SelfMLP models on (**a**) raw dataset and (**b**) segmented dataset.

Supplementary Figures S1, S3, S5, S7, S9 and S11 are the confusion matrix of MobileNetV2- LSTM- SelfMLP (q1), MobileNetV2-LSTM- SelfMLP (q3), MobileNetV2-LSTM-SelfMLP (q5), ResNet18-LSTM-SelfMLP (q1), ResNet18-LSTM-SelfMLP (q3), and ResNet18-LSTM-SelfMLP (q5), respectively, on the raw dataset, and also, Supplementary Figures S2, S4, S6, S8, S10 and S12 are the confusion matrix of same six configurations on the segmented dataset. From Supplementary Figures S1, S3, S5, S7, S9 and S11, it is apparent that ResNet18-LSTM-SelfMLP (q3) misclassified ‘Class 007’ as ‘Class 001’ on the raw dataset for the highest instances of 70. On the other hand, MobileNetV2-LSTM-SelfMLP (q3) is reported with 75 misclassification cases for predicting 75 Class 024 segmented videos as Class 009. However, MobileNetV2-LSTM-SelfMLP (q3) on the segmented dataset showed the highest misclassification of a single class. This classifier showed better classification performance than the other eleven classifiers (q = 1, 3, 5 based MobileNetV2-LSTM- SelfMLP and ResNet18-LSTM-SelfMLP classifiers) on the raw and segmented datasets. Figure 9a represents the ROC curves of MobileNetV2- and ResNet18-based on six classifiers. Among all classifiers, the highest AUC of 0.94 was found for MobileNetV2-LSTM-SelfMLP (q3), while the lowest AUC of 0.85 was reported for ResNet18-LSTM-SelfMLP (q1). So, the MobileNetV2-LSTM-SelfMLP (q3) model demonstrated the best among six models of Figure 9a in true-positive prediction over false-positive prediction.

The ROC curves of six classifiers on the segmented dataset are given in Figure 9b. The best AUC 0.99 is achieved by MobileNetV2-LSTM-SelfMLP (q3). All the architectures with q value of 1, 3, and 5 of ResNet-LSTM-SelfMLP achieved the lowest AUC among the six classifiers. Comparing Figure 9a,b, it is evident that MobileNetV2-LSTM-SelfMLP (q3) on the segmented dataset is the best classifier that has the ability on predicting most true-positive instances over false-positive instances.

### 4.1. Performance Evaluations of Models on Raw Dataset

Table 3 illustrates the accuracy of Arabic Sign Language recognition on the raw dataset in signer-independent mode. Sign Language Recognition in signer-independent mode is more challenging compared to signer-dependent mode [16]. Utilizing raw video for sign language recognition has been investigated in previous studies [17,46,47]. Among all six models in Table 3, the highest individual accuracy was achieved using MobileNetV2-LSTM-SelfMLP (q = 3) for Signer 1 and Signer 4. Moreover, the best performance was achieved for Signer 2 and Signer 3 using MobileNetV2-LSTM-SelfMLP (q = 5) and MobileNetV2-LSTM-SelfMLP (q = 1), respectively. On the other hand, models with ResNet18 spatial feature extractors showed lower performance compared to the counterpart models with MobileNetV2. It is also noticeable in Table 3 that MobileNetV2-LSTM-SelfMLP with q = 5 showed the smallest inference time of 158 milliseconds. Overall, the best average accuracy is reported on the raw dataset with 62.90% using MobileNetV2-LSTM-SelfMLP (q = 3).

The numerical evaluation of the six models is tabulated in Table 4. As illustrated, MobileNetV2-LSTM-SelfMLP (q = 3) showed the best overall accuracy, recall, F1 score, and specificity, while MobileNetV2-LSTM-SelfMLP (q = 3) performed the best precision among all six models. Although ResNet18-LSTM-SelfMLP with q = 1, 3, 5 have more trainable parameters compared to MobileNetV2-LSTM-SelfMLP-based models, the prior models are less accurate and precise in recognizing Arabic Sign Languages. The D1 score is not reported in Table 4 for ResNet18-LSTM-SelfMLP (q = 1) as the model failed to recognize all the sign videos of Classes 0035 and 0008, which is evident in Supplementary Figure S5. The same result is found for ResNet18-LSTM-SelfMLP (q = 5) as this model misclassified all the sign videos of Class 0002 as all other classes. Performance wise, ResNet18-LSTM-SelfMLP (q = 1) and ResNet18-LSTM-SelfMLP (q = 5) are the least performing dynamic sign Language recognition over raw videos.

### 4.2. Performance Evaluations of Models on Segmented Dataset

Sign language can be understood through facial expressions and hand shapes. Numerous methods have been applied to face and dominant hand region-based sign language recognition systems [48,49,50,51]. However, in this study, we highlighted the importance of segmented videos compared to raw videos for sign recognition. Six models with similar configurations, hyper-parameters, and trainable parameters were used to train over the raw and segmented data. All models showed improved performance for Arabic Sign Language recognition when trained on segmented data while showing inferior results with raw unsegmented data (Table 5). The segmentation guides the deep learning model to learn from ROI, face, and hand regions, avoiding irrelevant features from the background.

The best individual accuracy was achieved using MobileNetV2-LSTM-SelfMLP with q = 3 for Signer 1, Signer 2, and Signer 3. MobileNetV2-LSTM-SelfMLP (q = 1) performed the best on unseen Signer 4 testing data with 81.48% accuracy, while the second best accuracy of 80.47% was achieved by MobileNetV2-LSTM-SelfMLP with q = 3. Although ResNet18-LSTM-SelfMLP (q = 5) has the highest trainable parameters, it showed the lowest performance with 59.03% accuracy compared to its q = 1 and q = 3 counterparts. A similar trend was found in MobileNetV2-LSTM-SelfMLP for the segmented dataset where the model with q = 5 performed worse than the other variants with q = 1 and q = 3. It can be noticed that, due to their shallow architecture, MobileNetV2-LSTM-SelfMLP (q = 1) is the fastest with minimum inference time.

The motivation for region-based segmented dynamic sign language recognition was to improve the accuracy in signer-independent mode. In comparisons of Table 3 and Table 5, the face and hand region-based segmented approach outperformed the raw video approach by 34.97% in average accuracy. Additionally, this approach is more precise than the raw video approach to recognizing true-positive dynamic sign video which is reported in Table 6. The highest precision, recall, F1 score and specificity reported were 88.57%, 87.69%, 87.72%, and 99.75% respectively using MobileNetV2- LSTM-SelfMLP (q = 3). The variants of the MobileNetV2 spatial feature extractor with different *q* values performed better than the ResNet18 feature extractor models. Consequently, inverted residual structure and linear bottleneck layers [39] in MobileNetV2 architecture, which are alternatives to traditional residual models [40], performed best in this study. Additionally, the demonstration of improving performance by removing non-linearities in narrow layers [39] also helped in dynamic sign language recognition. Moreover, MobileNetV2 and ResNet18 models with q = 3 SelfMLP showed the best performance among other q values models. Although q = 5 has a much more complex architecture with additional nodal operations, it seems that it overfits for the targeted problem resulting in lower recognition performance. A similar result was found in [52,53] where the 1D SelfONN model with q = 3 showed better results compared to its counterpart with q = 5. By considering the overall accuracy, MobileNetV2-LSTM -SelfMLP (q = 3) is the best model in signer-independent mode for Arabic Sign Language recognition with 87.69% accuracy.

As the best accuracy was found by MobileNetV2-LSTM-SelfMLP (q = 3), a more rigorous way of performance evaluation is needed. Confidence intervals were used to quantify the uncertainty in the accuracy of the MobileNetV2-LSTM-SelfMLP (q = 3) model on the Arabic Sign Language video dataset. Table 7 represents the confidence interval of MobileNetV2-LSTM-SelfMLP (q = 3) at different percentiles. The model achieved an accuracy of 87.69%, with a confidence interval of 95%. This means that there is a 95% probability that the true accuracy of the model is within the range of 86.90% to 88.47%. The z-score for a 95% confidence interval is 1.96. This means that the standard error of the estimate is 0.40%. The standard error of the estimate is a measure of how much variation there is in the accuracy of the model. It is calculated by taking the square root of the variance of the accuracy. The confidence interval for different percentiles can be calculated by adding and subtracting the z-score for the desired percentile from the estimated accuracy. For example, the 90th percentile confidence interval is 87.02% to 88.35%, and the 99th percentile confidence interval is 86.65% to 88.73%. The confidence intervals found in this study suggest that the MobileNetV2-LSTM-SelfMLP (q = 3) model is a reliable and accurate model for Arabic Sign Language video classification. The narrow confidence intervals indicate that the true accuracy of the model is likely to be close to the estimated accuracy. However, this study is the first to use confidence intervals to quantify the uncertainty in the accuracy of these models, which was not reported in [16]. The use of confidence intervals provides a more informative way to assess the performance of deep learning models for Arabic Sign Language video classification.

### 4.3. Comparative Analysis on Arabic Sign Language Recognition Systems

**Figure 10:**
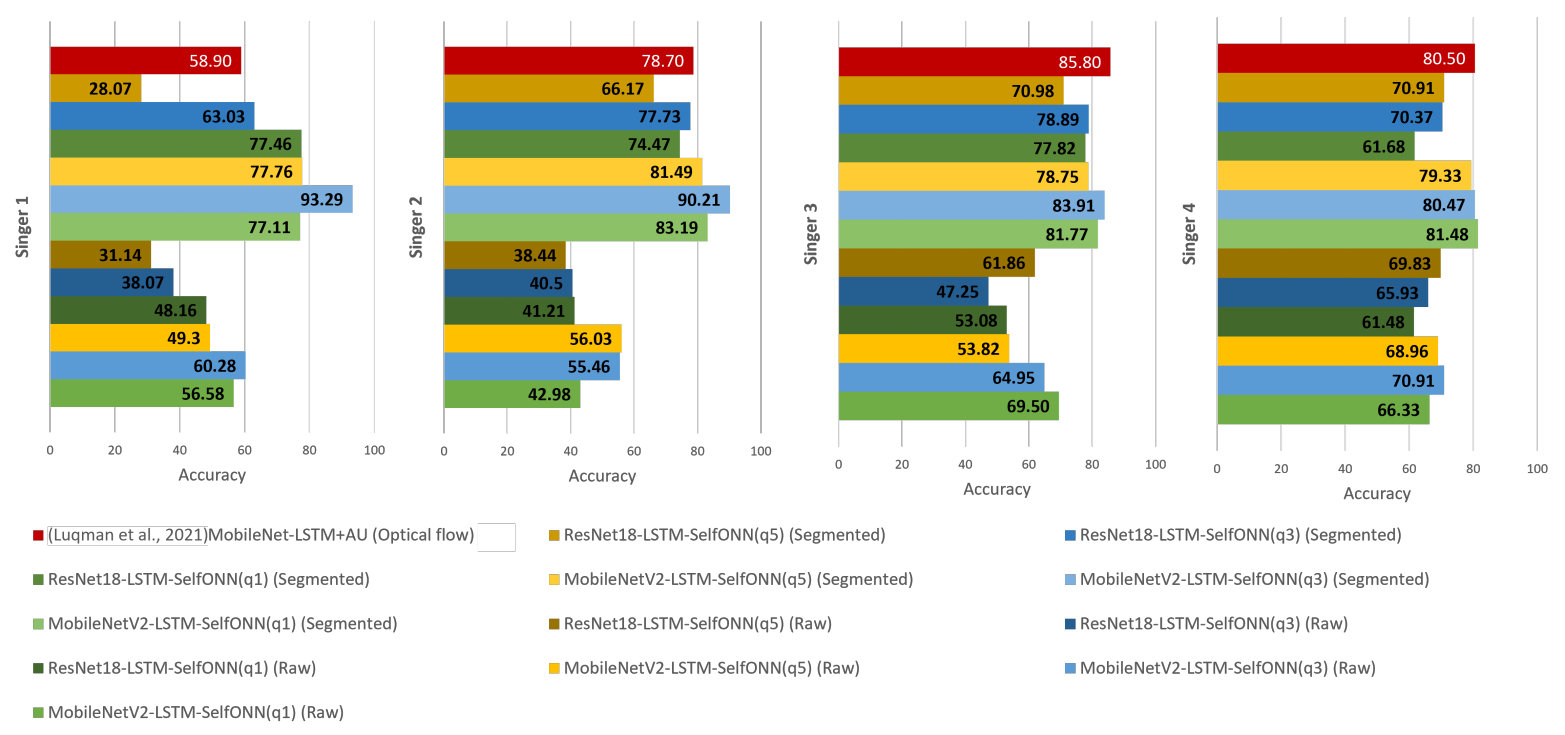
Signer-independent accuracy comparison of twelve CNN-LSTM-SelfMLP models on raw and segmented datasets with counterpart literature [16].

A comparative accuracy analysis of signer-independent Arabic Sign Language is shown in Figure 10. The six models trained on raw and segmented dataset for four signers are compared with previously published results in literature [16]. In [16], the authors found the best result on optical flow dataset rather than only raw (color) dataset. Animation units (AUs) of the signer face provided by Kinect sensors were fused with the MobileNetV2-LSTM model architecture in [16], achieving 76.00% average accuracy. In our study, MediaPipe Holistic was used for hand-face region segmentation and MobileNetV2-LSTM-SelfMLP (q = 3) achieved the best average accuracy of 86.97% which outperforms the best performance reported in [16].

The previous study used a technique that fused animation units (AUs) of signer face with MobileNetV2-LSTM model architecture. This technique had several limitations. First, it required the use of a Kinect sensor, which is not always available. Second, the AUs were not always accurate, which could lead to errors in the classification results. Third, the technique did not take into account the variability in sign language use among individuals.

Our study used a different technique that involved using MediaPipe Holistic to segment the hands and faces of the signers in the videos. This technique has several advantages. First, it does not require the use of any special hardware. Second, it is more accurate than the AU-based technique. Third, it can be used to segment the hands and faces of signers from different backgrounds and viewpoints. This segmented approach with SelfMLP integration in CNN-LSTM architecture increased the accuracy by 10.97% on average. Furthermore, MobileNetV2-LSTM-SelfMLP (q = 3) outperformed literature [16] in individual Signer 1 and Signer 2 accuracy with a large margin as shown in Figure 10. The best approach adopted in this study increased with 34.39% and 11.51% independent test accuracy for Signer 1 and Signer 2, respectively. Additionally, MobileNetV2-LSTM-SelfMLP (q = 3) achieved close accuracy levels for Signer 4 but only 0.03% less than literature [16]. On the other hand, MobileNetV2-LSTM-SelfMLP (q = 1) achieved the best individual result for Signer 4, 81.48%, with a0.98% improvement over previously reported result 80.50% in [16]. In the Signer 3 independent testing, the best-performing MobileNetV2-LSTM-SelfMLP (q = 3) achieved 83.91% accuracy, while a previous investigation in [16] showed 85.80% accuracy. In both investigations, state-of-the CNNs were used as feature extractors. In this study, two LSTM layers were used, similar to what has been used in previous literature studies. However, the number of hidden layer neurons was different. In [16], 1024 neurons were used for each LSTM layer, while in this study, more compact LSTM layers with 310 neurons were used. Subsequently, the SelfMLP classifier helped to enhance the performance compared to conventional MLP layers. Finally, the MobileNetV2-LSTM-SelfMLP (q = 3) architecture showed generally the best ever achieved performance for Arabic Sign Language recognition.

The proposed MobileNetV2-LSTM-SelfMLP (q = 3) model on the segmented dataset outperforms other contemporary literature in the Arabic Sign Language domain and a comparative analysis is given in Table 8. In [32], the authors used a 2D convolutional recurrent neural network (2DCRNN) and a 3D convolutional neural network (3DCNN) for Arabic Sign Language recognition. The best accuracy of 99% was achieved using a 3DCNN, but the model was heavier and had a slower inference time than our proposed model. The authors also used a dataset of 56 classes, while we used a dataset of 50 classes. Additionally, the dataset used in [32] only had 224 videos, which is a small number for training a deep learning model. In contrast, we used a four-fold cross-validation with signer-independent mode, which means that we tested our model on unseen data from four different signers. Each signer had a different number of videos: 455, 547, 544, and 543, respectively.

Another study [35] used a larger dataset of 100 classes and pose-based transformers for Arabic Sign Language recognition. The authors also used signer-independent mode for evaluation, as we did. However, they only achieved an accuracy of 68.2%, while we achieved 87.69%. Additionally, the KArSL-100 dataset used in [35] was recorded from only three subjects, while our dataset had data from four subjects. This suggests that our proposed model is more robust to variations in signers’ hand movements. Overall, the results of our study demonstrate that the proposed MobileNetV2-LSTM-SelfMLP (q = 3) model outperforms other contemporary literature in the Arabic Sign Language domain.

## 5. Conclusions and Future Work

The implementation of SelfMLP-infused CNN-LSTM models on a multi-modality video dataset for Arabic Sign Language recognition is presented in the proposed work. Two cases such as the raw (color) dataset and segmented dataset were considered for signer-independent test modes using MobileNetV2- and ResNet18-based CNN-LSTM-SelfMLP architectures. Raw (color) dataset videos were used at first, and then MediaPipe Holistic based face–hand region segmented datasets were used. To estimate the performance for finding out the best Arabic Sign Language recognition model, six models were trained in both datasets. Transfer learning and fine-tuning methods were used on MobileNetV2 and ResNet18 for spatial feature extraction. The best model MobileNetV2-LSTM-SelfMLP (q = 3) was found on the segmented dataset. It attained 86.97% average accuracy and 87.69% overall accuracy with outstanding improvement in manual and non-manual sign language recognition compared to the previously reported study on the same dataset [16]. The use of self-MLP with q in the order of 3 benefited the model to boost the accuracy.

The daily life of a sign language user is full of difficulties when it comes to communicating with non-signer people in various aspects such as transportation, medicine, education, or even shopping as well. Therefore, a system needs to be developed to reduce the problem of this deaf and hard-of-hearing community. One limitation of this study is that the dataset used for sign language classification has a limited number of subjects and classes. In the future, an Arabic Sign Language recognition system will be developed with the proposed technique for a larger dataset, which will include sign alphabets; numerals, words, and sentences from multiple signers; different backgrounds; different lighting conditions; and different camera angles. The future model trained on a robust dataset will be implemented as a sign language transformer for the task related to a conversation between signer patients and doctors [6] and other scenarios. For a full-scale implementation of the sign language transformer reported in [6], the proposed technique will be investigated with landmark data from MediaPipe Holistic instead of videos as input. To develop the sign language transformer reported in [6], we will also develop the necessary hardware implementation. The attention mechanism has recently gained popularity in action recognition, gesture recognition, and other computer vision tasks. This is because attention allows the model to focus on the most relevant parts of the input, which can improve the accuracy of the model. In our future work, we plan to integrate the attention mechanism into our model architecture for Arabic sign video classification. This will allow us to train our model on larger datasets and to recognize sign videos in real-world scenarios. We also aim to implement other new state-of-the-art 1D and 2D CNNs in a real-time Arabic Sign Language Transformer design in the near future.

References yes

## Figures and Tables

**Table 1:** Details of training parameters of MobileNetV2 and ResNet18.

Training Parameters	MobileNet_V2-LSTM-SelfMLP	ResNet18-LSTM-SelfMLP
LSTM Hidden Size	310	310
LSTM Layer	2	2
Input Dimension	224×224×3	224×224×3
Frames per Video	20	20
Batch Size	3	3
Learning Rate	0.0001	0.0001
Max Epochs	50	50
Epoch Patience	6	6
Learning Rate Drop factor	0.2	0.2
Epoch Stopping Criteria	10	10
Optimizer	Adam	Adam

**Table 2:** Details of number of trainable parameters of six models used in Arabic Sign Language recognition.

Model	q = 1	q = 3	q = 5
MobileNetV2-LSTM-SelfMLP	4,980,834	5,003,874	5,026,914
ResNet18-LSTM-SelMLP	12,981,154	13,004,194	13,027,234

**Table 3:** Independent signer test accuracy on Arabic Sign Language raw dataset using MobileNetV2- and ResNet18-based CNN+LSTM+SelfMLP models. Light gray background indicates best performance.

Model	q	Inference Time (s)	Tested Signer				
			Signer 1	Signer 2	Signer 3	Signer 4	Average Accuracy
MobileNetV2-LSTM-SelfMLP	1	0.159538963	56.58	42.98	69.5	66.33	58.848
	3	0.157735353	60.28	55.46	64.95	70.91	62.900
	5	0.157735061	49.3	56.03	53.82	68.96	57.028
ResNet18-LSTM-SelfMLP	1	0.071416741	48.16	41.21	53.08	61.48	50.983
	3	0.071358249	38.07	40.5	47.25	65.93	47.938
	5	0.071394228	31.14	38.44	61.86	69.83	50.318

**Table 4:** Overall performance analysis of MobileNetV2- and ResNet18-based six CNN-LSTM-SelfMLP models on the raw dataset. Light gray background indicates best performance.

Model	q	Overall Accuracy	Precision	Recall	F1 Score	Specificity
MobileNetV2-LSTM-SelfMLP	1	58.77	66.83	58.77	57.85	99.18
	3	62.62	66.04	62.62	61.68	99.24
	5	56.11	62.84	56.11	55.21	99.10
ResNet18-LSTM-SelfMLP	1	50.76	52.39	50.76		98.98
	3	46.84	53.35	46.84	43.37	98.92
	5	48.18	54.98	48.18		98.95

**Table 5:** Independent signer test accuracy on Arabic Sign Language segmented dataset using MobileNetV2- and ResNet18-based CNN-LSTM-SelfMLP models. Light gray background indicates best performance.

Model	q	Inference Time (s)	Tested Signer				
			Signer 1	Signer 2	Signer 3	Signer 4	Average Accuracy
MobileNetV2-LSTM-SelfMLP	1	0.159056542	77.11	83.19	81.77	81.48	80.888
	3	0.159376328	93.29	90.21	83.91	80.47	86.970
	5	0.160681521	77.76	81.49	78.75	79.33	79.333
ResNet18-LSTM-SelfMLP	1	0.071896866	77.46	74.47	77.82	72.53	75.570
	3	0.071335469	63.03	77.73	78.89	70.37	72.505
	5	0.071379997	52.50	66.17	70.98	70.91	59.033

**Table 6:** Overall performance analysis of MobileNetV2- and ResNet18-based six CNN-LSTM-SelfMLP models on segmented dataset. Light gray background indicates best performance.

Model	q	Overall Accuracy	Precision	Recall	F1 Score	Specificity
MobileNetV2-LSTM-SelfMLP	1	80.41	83.37	80.41	80.10	99.61
	3	87.69	88.57	87.69	87.72	99.75
	5	79.12	80.37	79.12	78.66	99.57
ResNet18-LSTM-SelfMLP	1	75.81	78.48	75.81	75.09	99.50
	3	71.32	75.23	71.32	71.92	99.42
	5	63.63	62.36	55.27	56.16	99.06

**Table 7:** The confidence interval of MobileNetV2-LSTM-SelfMLP (q = 3) at different percentiles.

Percentile	Standard Error	$z_{\mathrm{score}}$	Lower Bound	Upper Bound
90th	0.40	1.65	87.02	88.35
95th	0.40	1.96	86.90	88.47
99th	0.40	2.58	86.65	88.73

**Table 8:** Comparative analysis of the results of previously published studies in Arabic Sign Language domain with the proposed model.

Literature	Technique	Number of Classes	Accuracy
[32]	2D convolutional recurrent neural network (2DCRNN)	56	92.00%
	3D convolutional neural network (3DCNN)	56	99.00%
[35]	Pose-based Transformer, KArSL-100 dataset	100	68.2%
	Pose-based Transformer, LSA64 dataset	64	91.09%
Our proposed study	MobileNetV2-LSTM-SelfMLP (q = 3), face and hand region segmentations	50	87.69

## Data Availability

The data that support the findings of this study are available from the corresponding author upon reasonable request.

## Supplementary Materials

The following supporting information can be downloaded at: https://www.mdpi.com/article/10.3390/s23167156/s1.
