# Supplementary material for: Signer-Independent Arabic Sign Language Recognition System Using Deep Learning Model
Source: Sensors (Basel). 2023 Aug 14;23(16):7156. doi: 10.3390/s23167156 (PMC10459624; doi:10.3390/s23167156)
Supplement: Supplementary file 1 [file sensors-23-07156-s001.zip › sensors-2493994-supplementary.pdf]

Model: MobileNetV2-LSTM-SelfMLP (q1) on Raw Dataset

The accuracy and loss curves during the training of **MobileNetV2-LSTM-SelfMLP (q1)** on Raw Dataset is given Table S1:

Table S1: Accuracy and Loss curves of MobileNetV2-LSTM-SelfMLP (q1) training on raw dataset

**Fold**	**Accuracy**	**Loss**
1	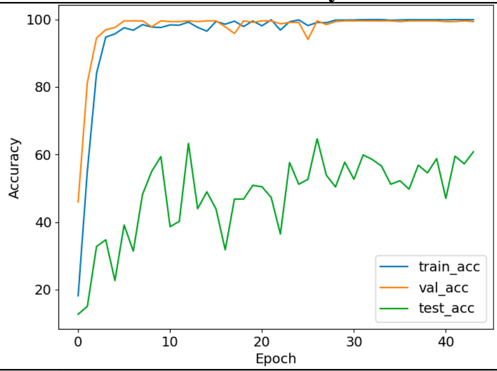	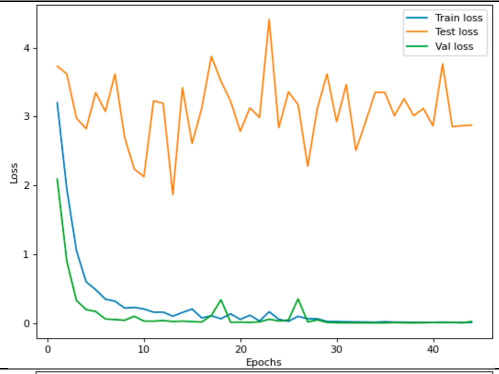
2	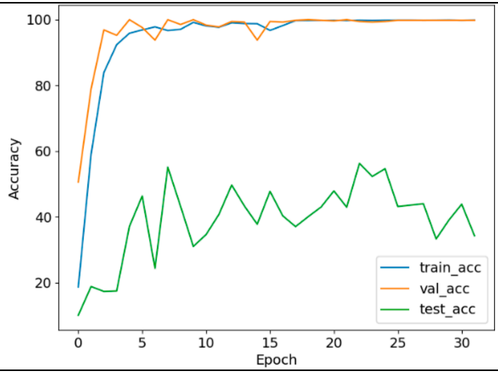	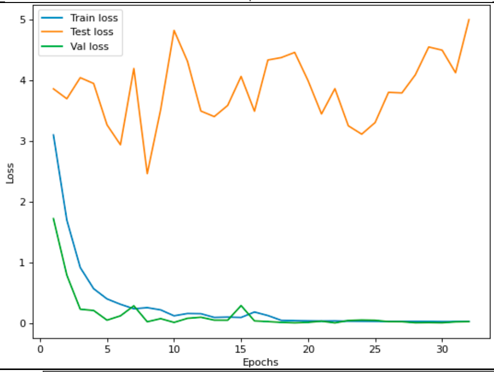
3	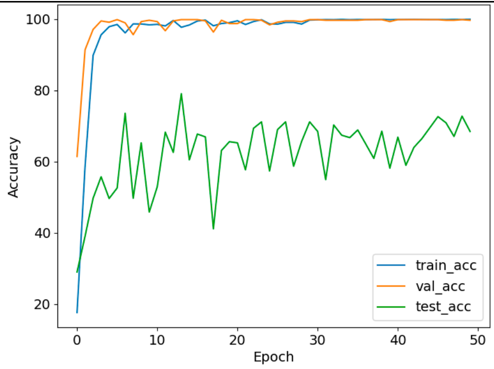	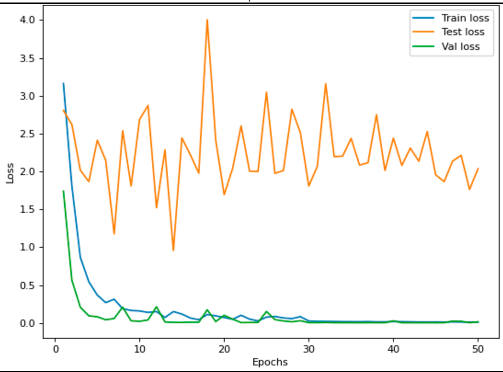

<table>
<tr>
<td>4</td>
<td>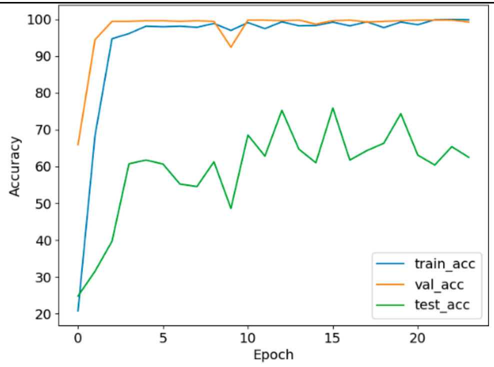
</td>
<td>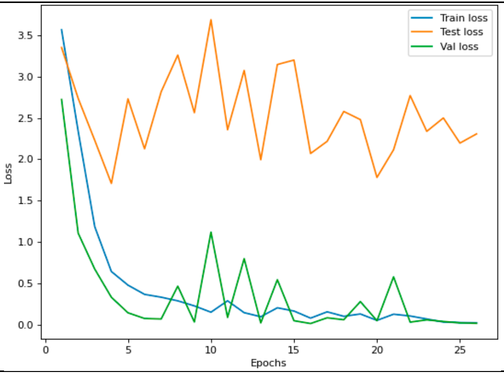
</td>
</tr>
</table>

Confusion Matrix: The Confusion Matrix of MobileNetV2-LSTM-SelfMLP (q1) on Raw Dataset is given below:

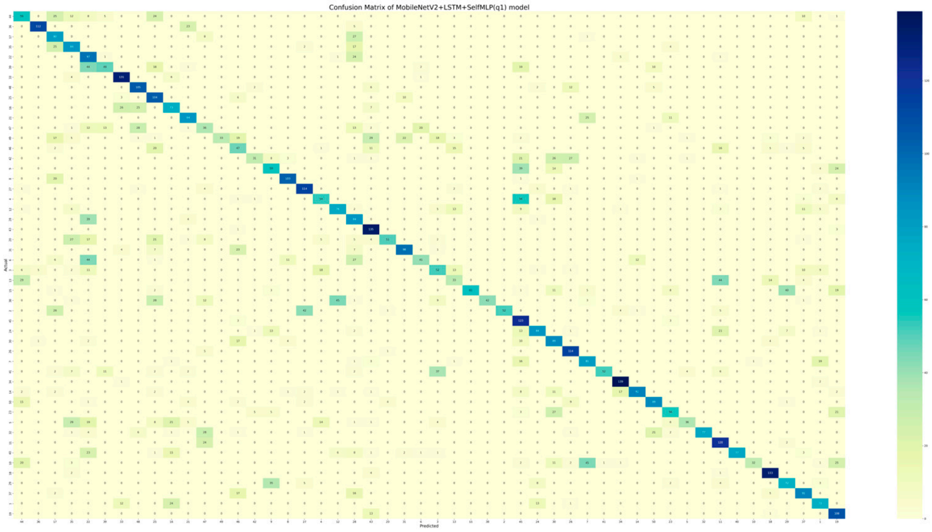

Figure S1: Confusion Matrix of MobileNetV2-LSTM-SelfMLP (q1) on Raw Dataset

ROC Curve: The ROC Curve of MobileNetV2-LSTM-SelfMLP (q1) on Raw Dataset is given below:

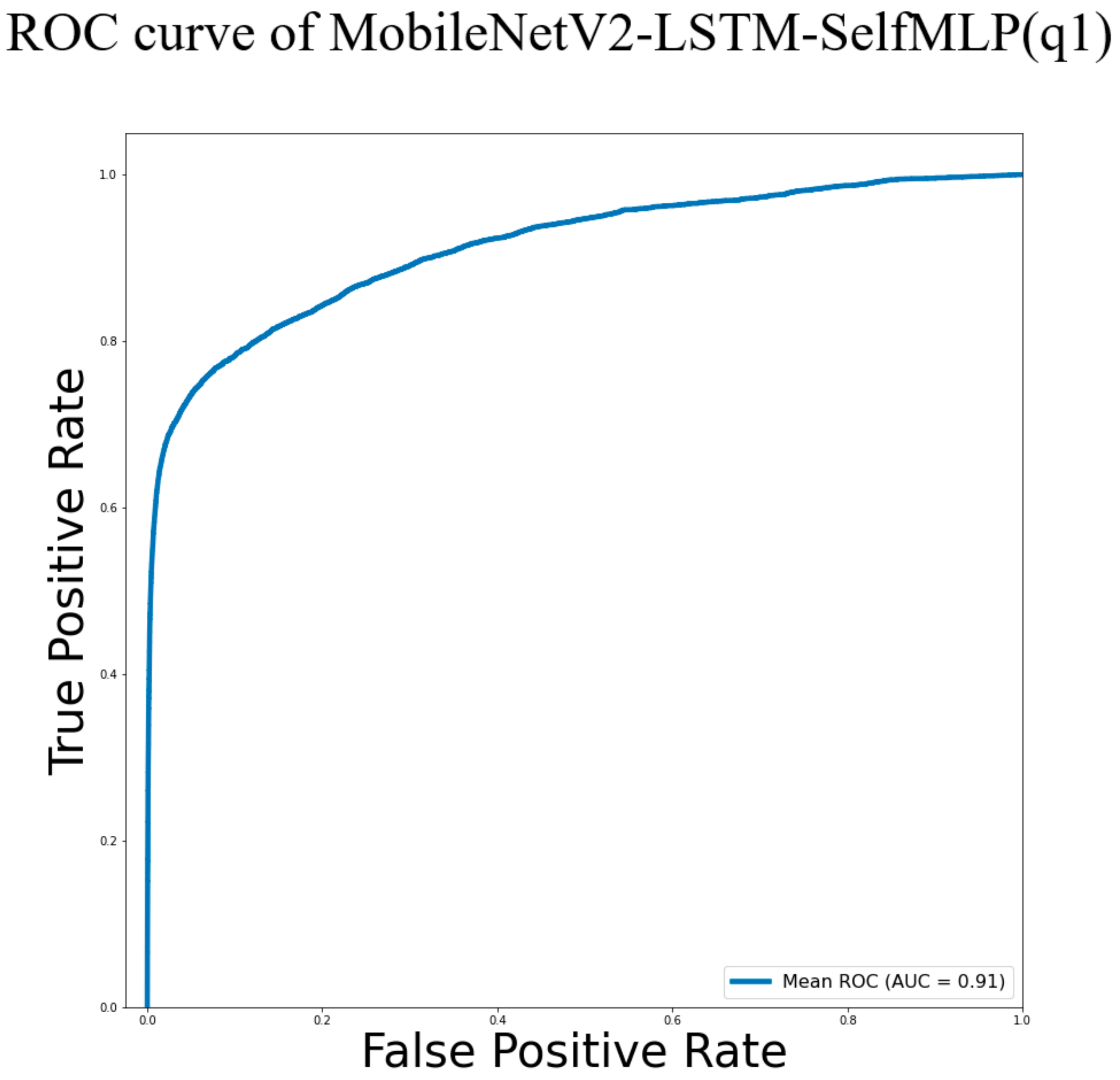

Figure S2: ROC Curve of MobileNetV2-LSTM-SelfMLP (q1) on Raw Dataset

Model: MobileNetV2-LSTM-SelfMLP (q3) on Raw Dataset

The accuracy and loss curves during the training of **MobileNetV2-LSTM-SelfMLP (q3)** on Raw Dataset is given Table S2:

Table S2: Accuracy and Loss curves of MobileNetV2-LSTM-SelfMLP (q3) training on raw dataset

**Fold**	**Accuracy**	**Loss**
1	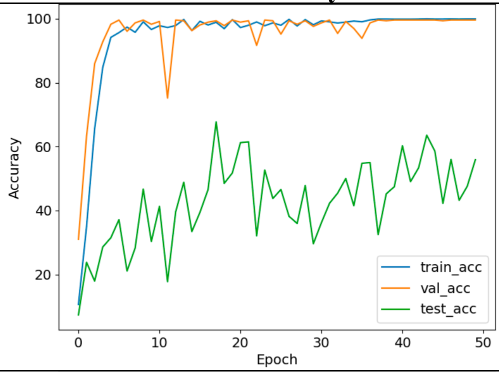	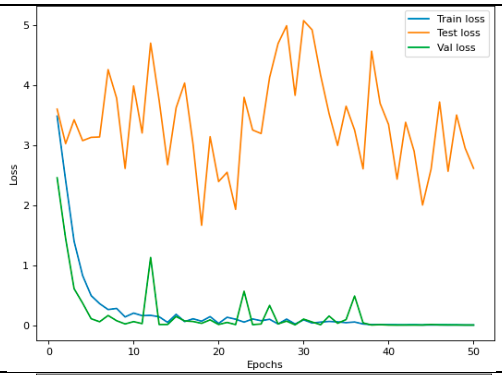
2	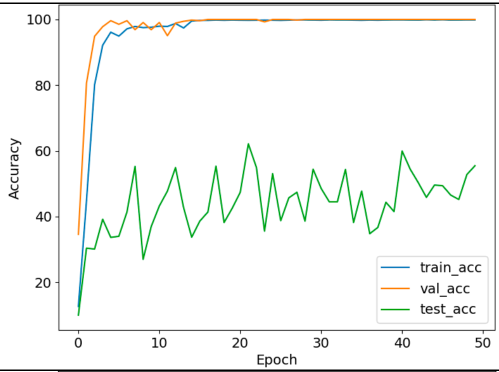	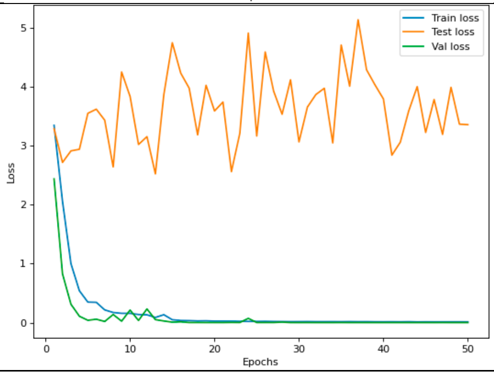
3	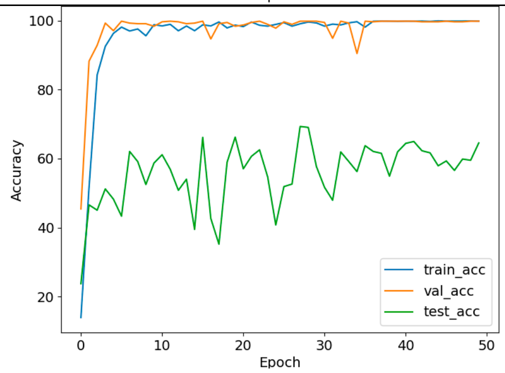	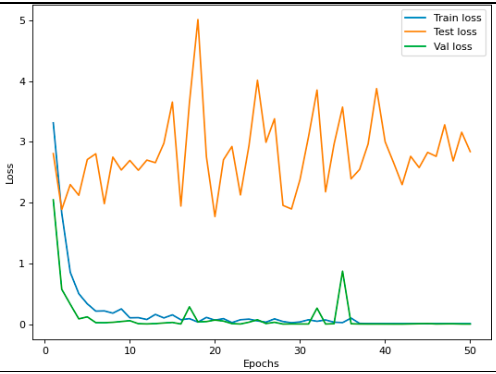

4	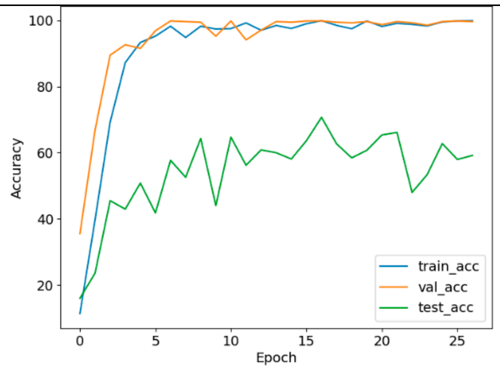train_acc<br>val_acc<br>test_acc<br>Accuracy<br>Epoch	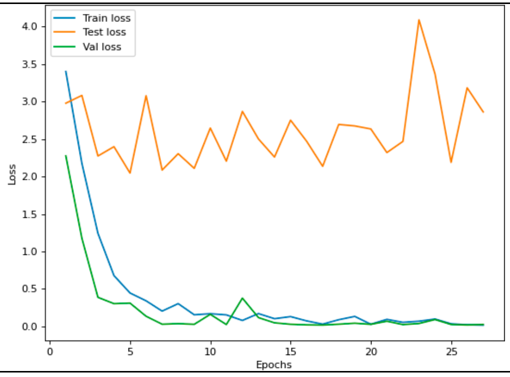Train loss<br>Test loss<br>Val loss<br>Loss<br>Epochs

Confusion Matrix: The Confusion Matrix of MobileNetV2-LSTM-SelfMLP (q3) on Raw Dataset is given below:

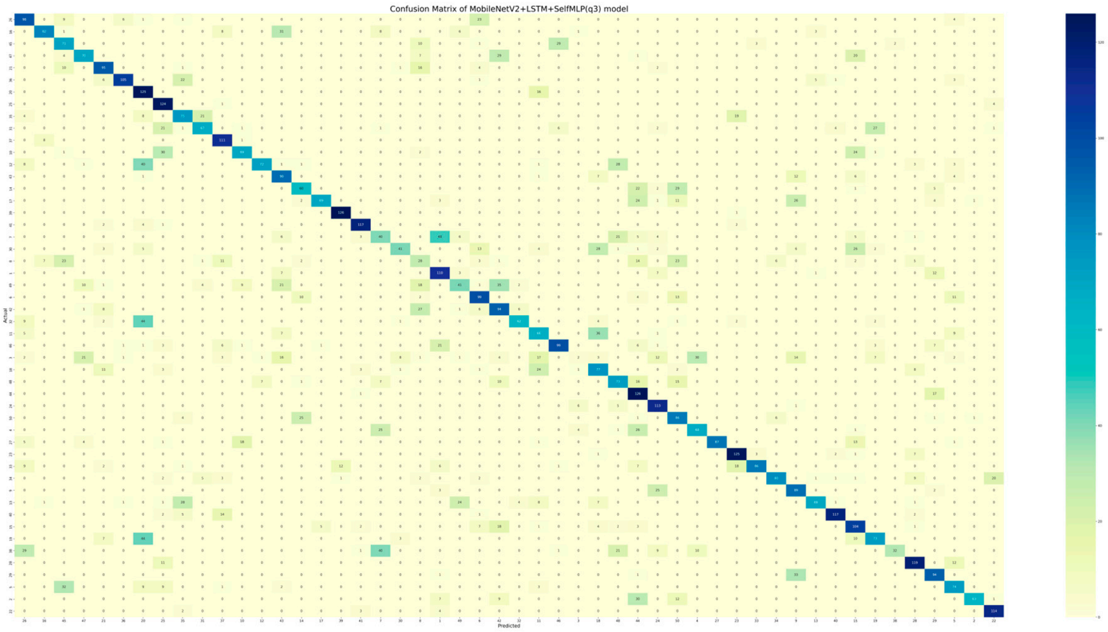

Figure S3: Confusion Matrix of MobileNetV2-LSTM-SelfMLP (q3) on Raw Dataset

ROC Curve: The ROC Curve of MobileNetV2-LSTM-SelfMLP (q3) on Raw Dataset is given below:

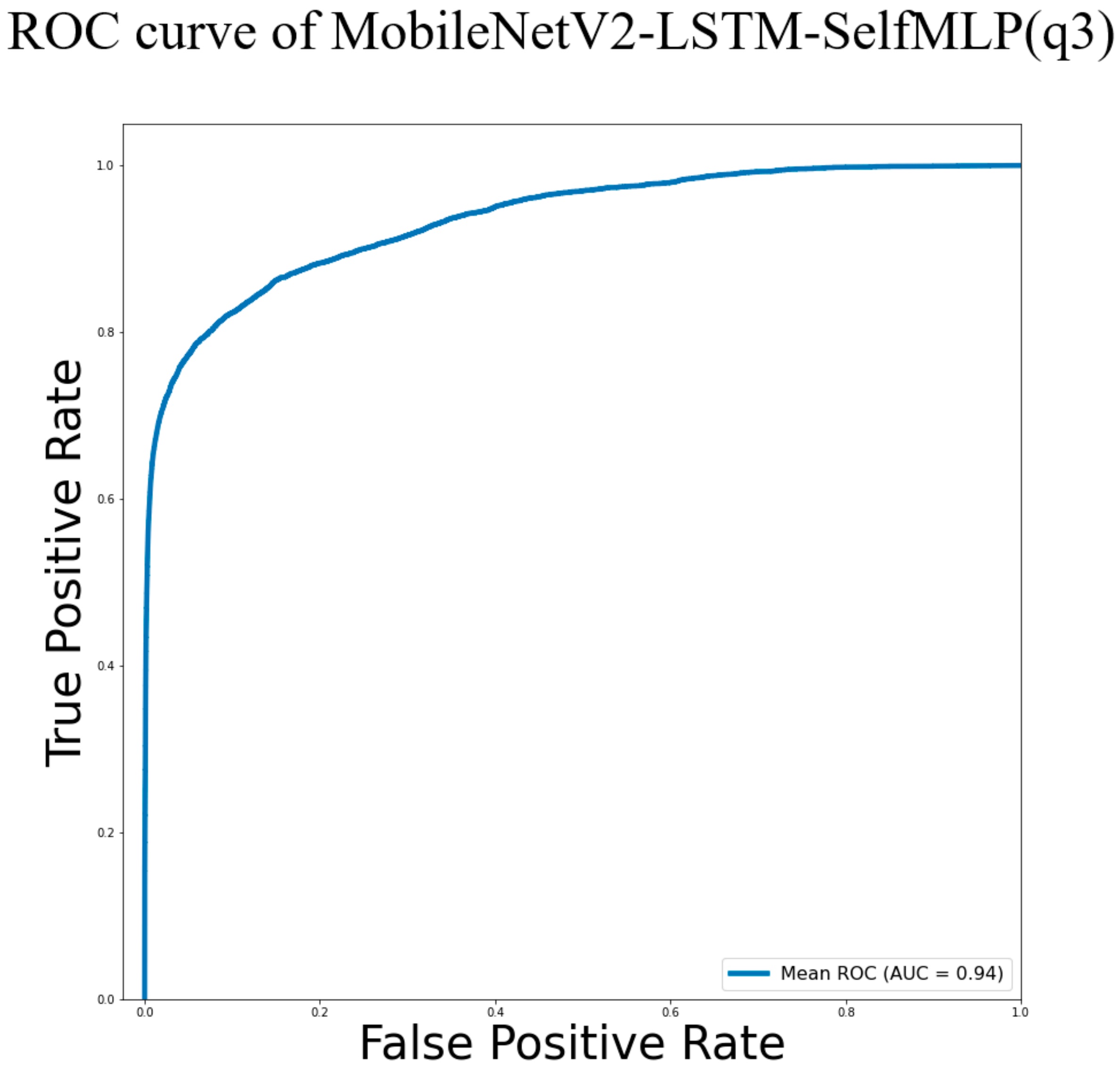

Figure S4: ROC Curve of MobileNetV2-LSTM-SelfMLP (q3) on Raw Dataset

Model: MobileNetV2-LSTM-SelfMLP (q5) on Raw Dataset

The accuracy and loss curves during the training of **MobileNetV2-LSTM-SelfMLP (q5)** on Raw Dataset is given Table S3:

Table S3: Accuracy and Loss curves of MobileNetV2-LSTM-SelfMLP (q3) training on raw dataset

Fold	Accuracy	Loss
1	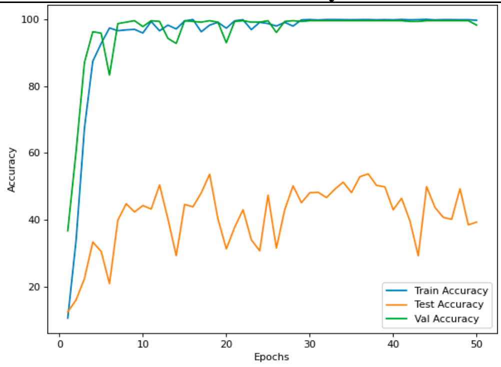	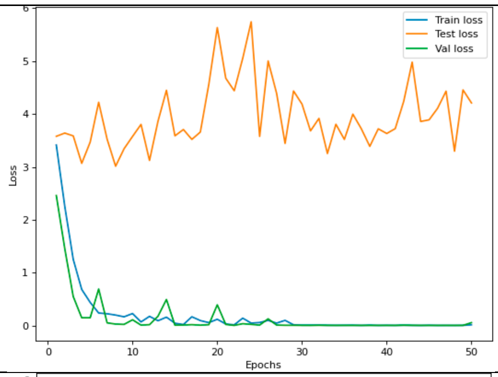
2	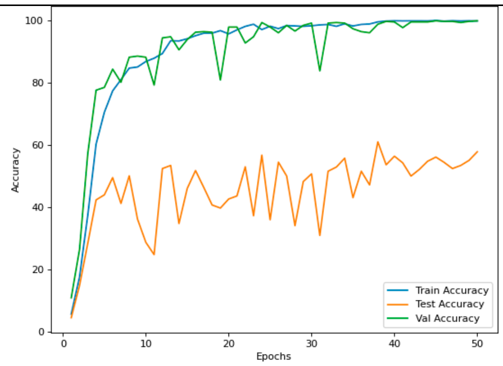	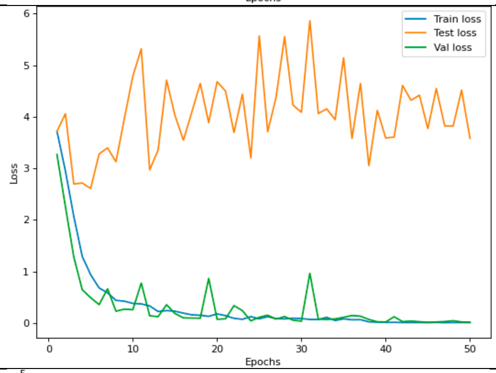
3	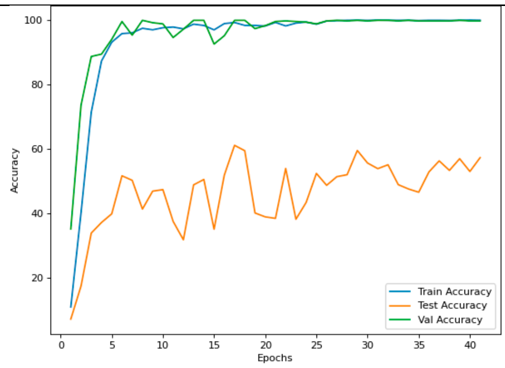	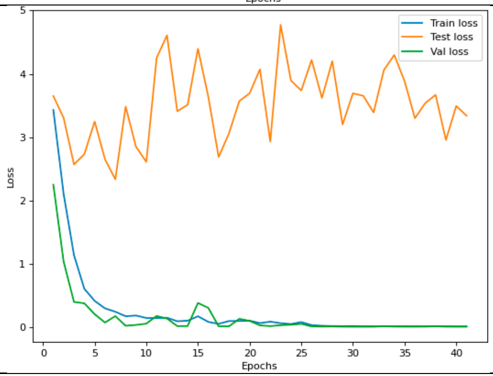

4

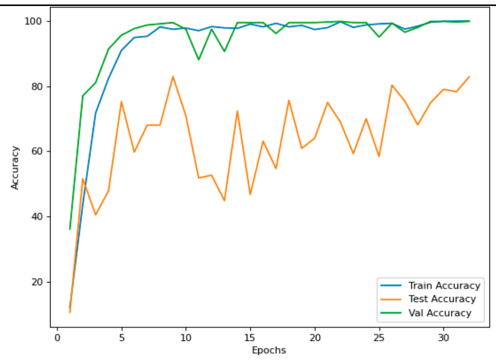
Accuracy
Epochs
Train Accuracy
Test Accuracy
Val Accuracy

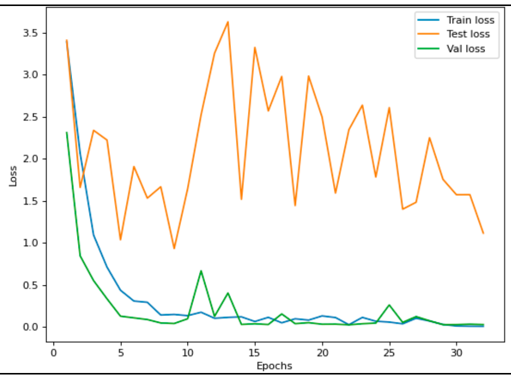
Loss
Epochs
Train loss
Test loss
Val loss

Confusion Matrix: The Confusion Matrix of MobileNetV2-LSTM-SelfMLP (q5) on Raw Dataset is given below:

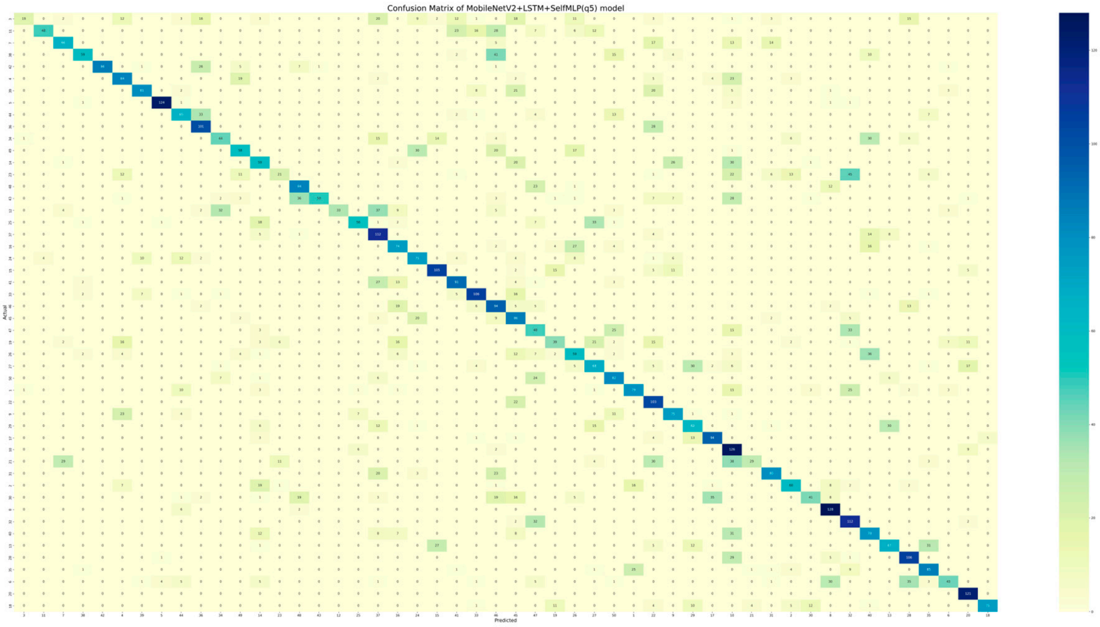

Figure S5: Confusion Matrix of MobileNetV2-LSTM-SelfMLP (q5) on Raw Dataset

ROC Curve: The ROC Curve of MobileNetV2-LSTM-SelfMLP (q5) on Raw Dataset is given below:

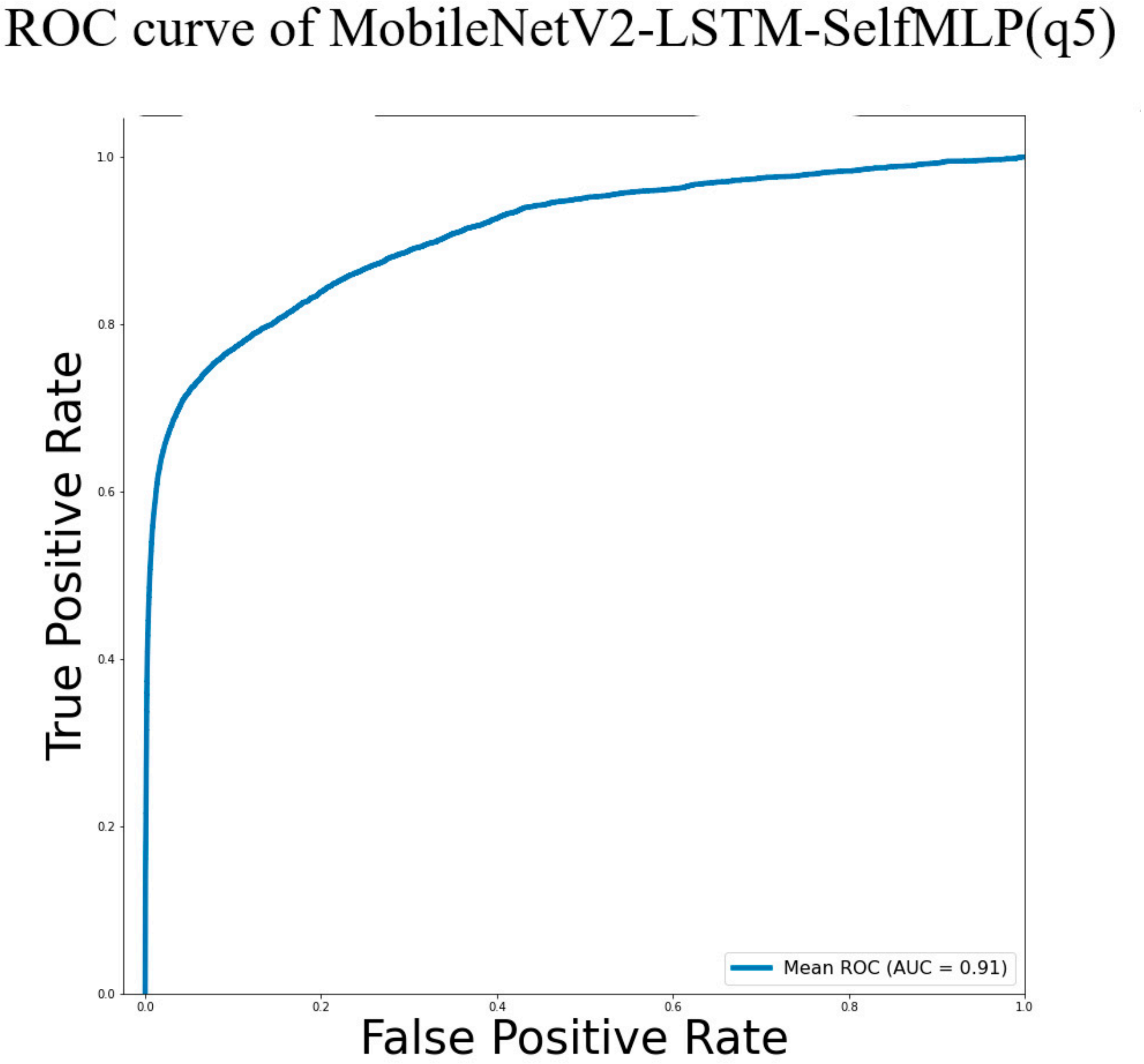

Figure S6: ROC Curve of MobileNetV2-LSTM-SelfMLP (q5) on Raw Dataset

Model: ResNet18-LSTM-SelfMLP(q1) on Raw Dataset

The accuracy and loss curves during the training of **ResNet18-LSTM-SelfMLP(q1)** on Raw Dataset is given Table S4:

Table S4: Accuracy and Loss curves of ResNet18-LSTM-SelfMLP(q1) training on raw dataset

<table>
<tr><th>Fold</th><th>Accuracy</th><th>Loss</th></tr>
<tr><td>1</td><td>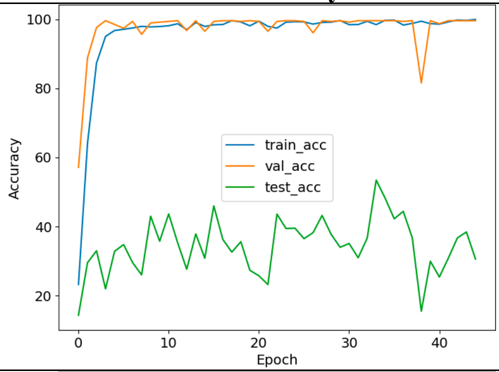
</td><td>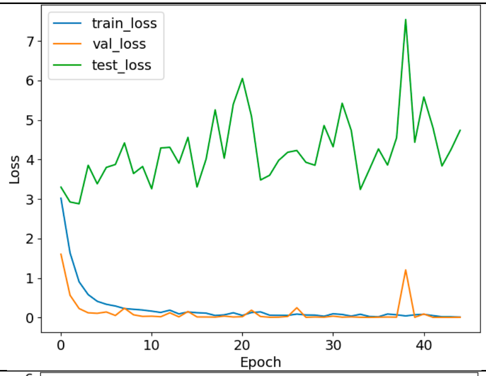
</td></tr>
<tr><td>2</td><td>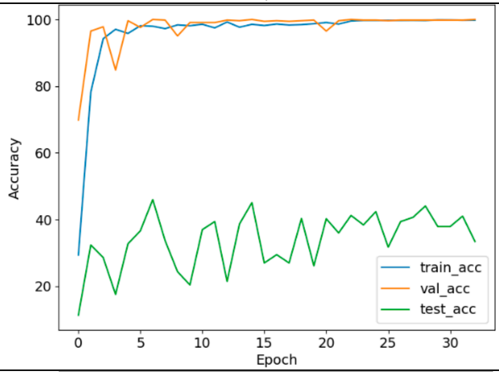
</td><td>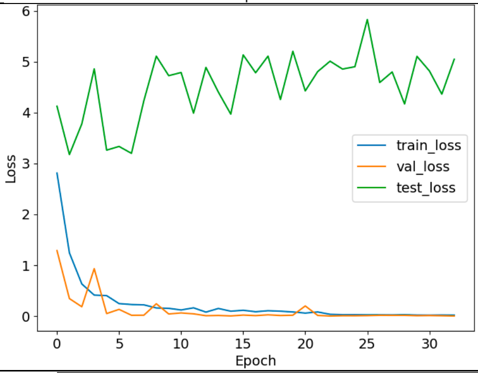
</td></tr>
<tr><td>3</td><td>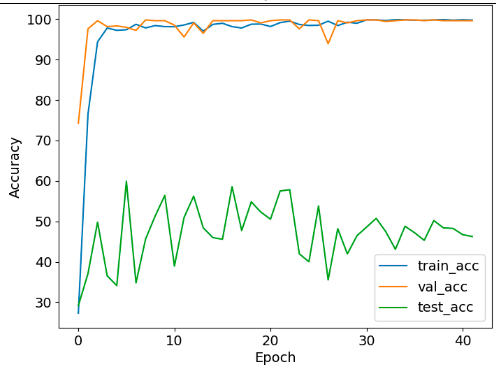
</td><td>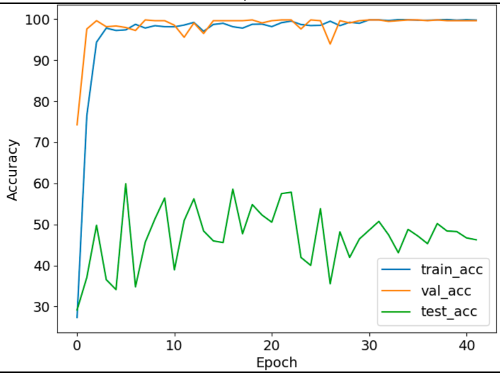
</td></tr>
</table>

4	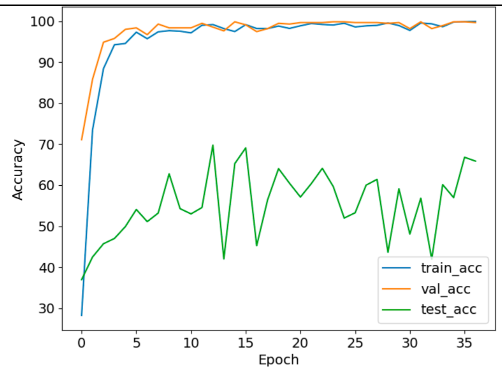 Accuracy<br>Epoch<br>train_acc<br>val_acc<br>test_acc	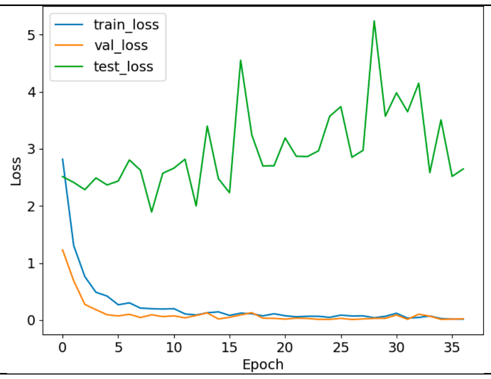 Loss<br>Epoch<br>train_loss<br>val_loss<br>test_loss

Confusion Matrix: The Confusion Matrix of ResNet18-LSTM-SelfMLP(q1) on Raw Dataset is given below:

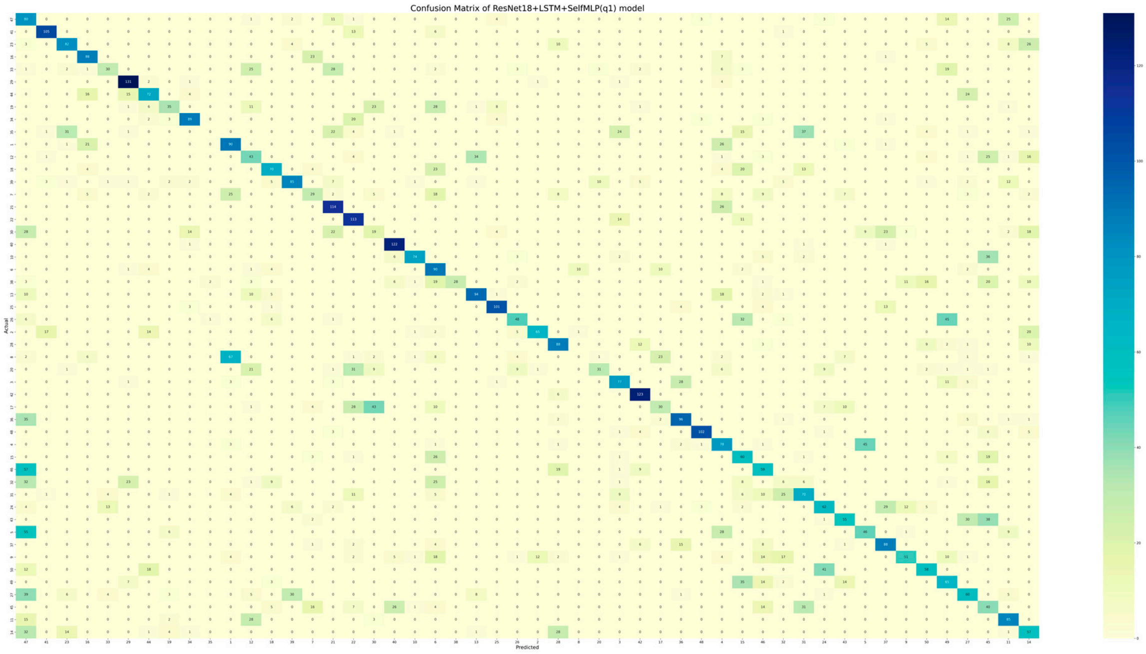

Figure S7: Confusion Matrix of ResNet18-LSTM-SelfMLP(q1) on Raw Dataset

ROC Curve: The ROC Curve of ResNet18-LSTM-SelfMLP(q1) on Raw Dataset is given below:

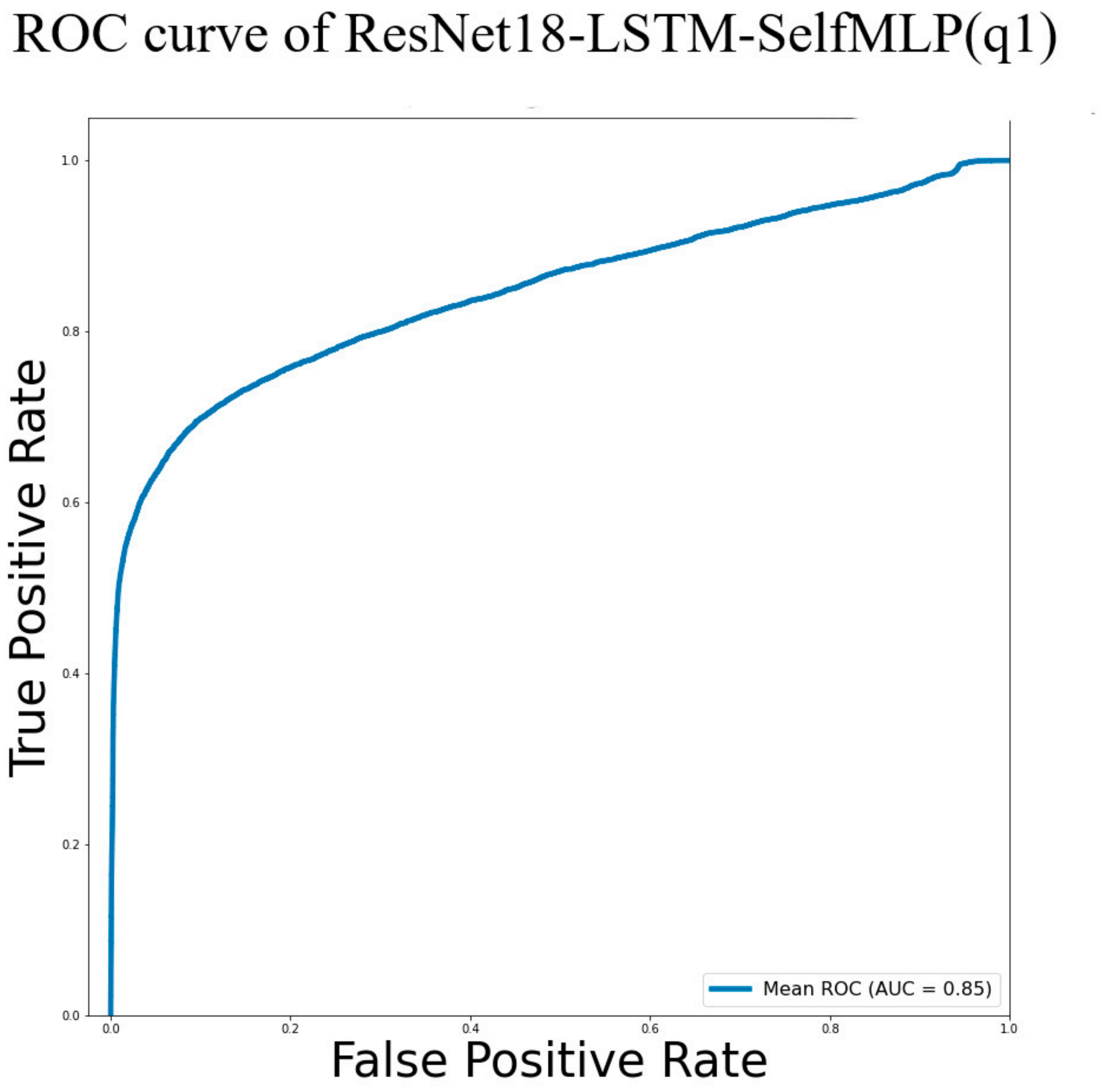

Figure S8: ROC Curve of ResNet18-LSTM-SelfMLP(q1) on Raw Dataset

Model: ResNet18-LSTM-SelfMLP(q3) on Raw Dataset

The accuracy and loss curves during the training of **ResNet18-LSTM-SelfMLP(q3)** on Raw Dataset is given Table S5:

Table S5: Accuracy and Loss curves of ResNet18-LSTM-SelfMLP(q3) training on raw dataset

Fold	Accuracy	Loss
1	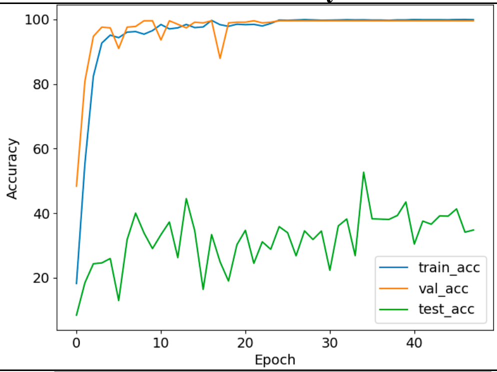	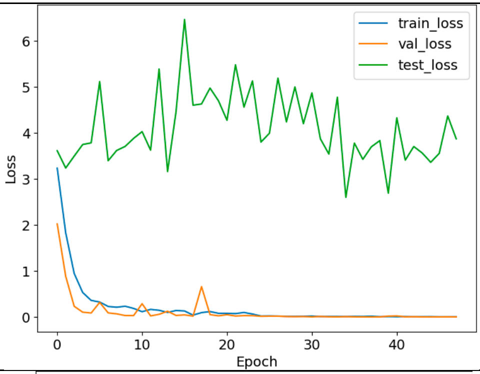
2	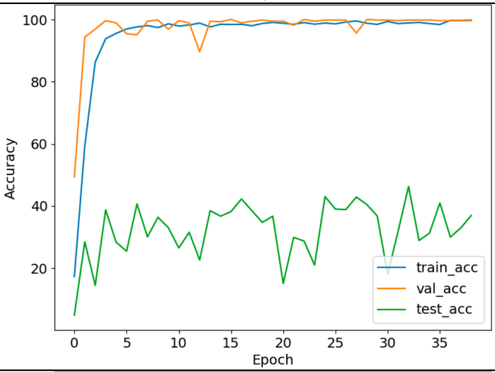	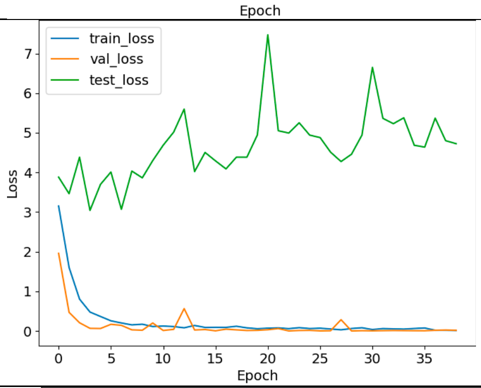
3	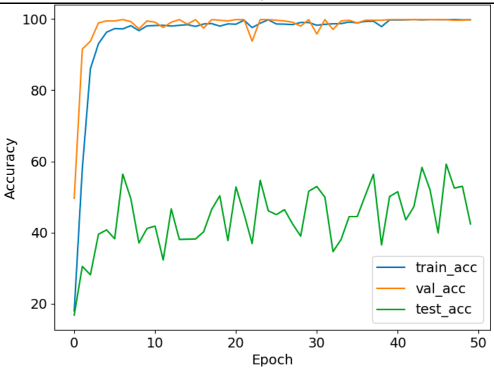	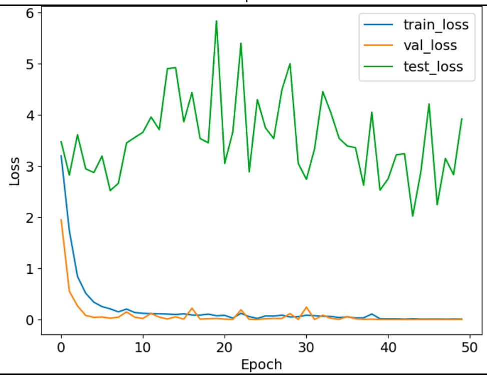

4	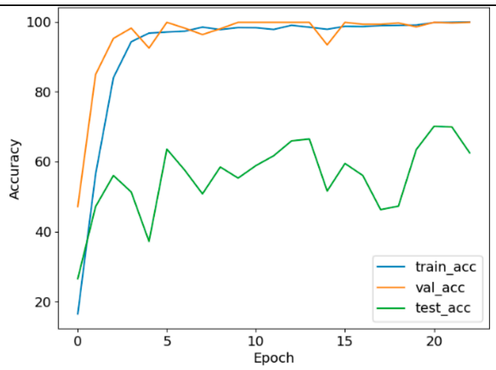Accuracy<br>Epoch<br>train_acc<br>val_acc<br>test_acc	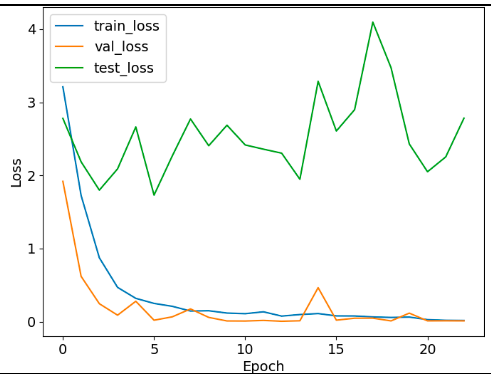Loss<br>Epoch<br>train_loss<br>val_loss<br>test_loss

Confusion Matrix: The Confusion Matrix of ResNet18-LSTM-SelfMLP(q3) on Raw Dataset is given below:

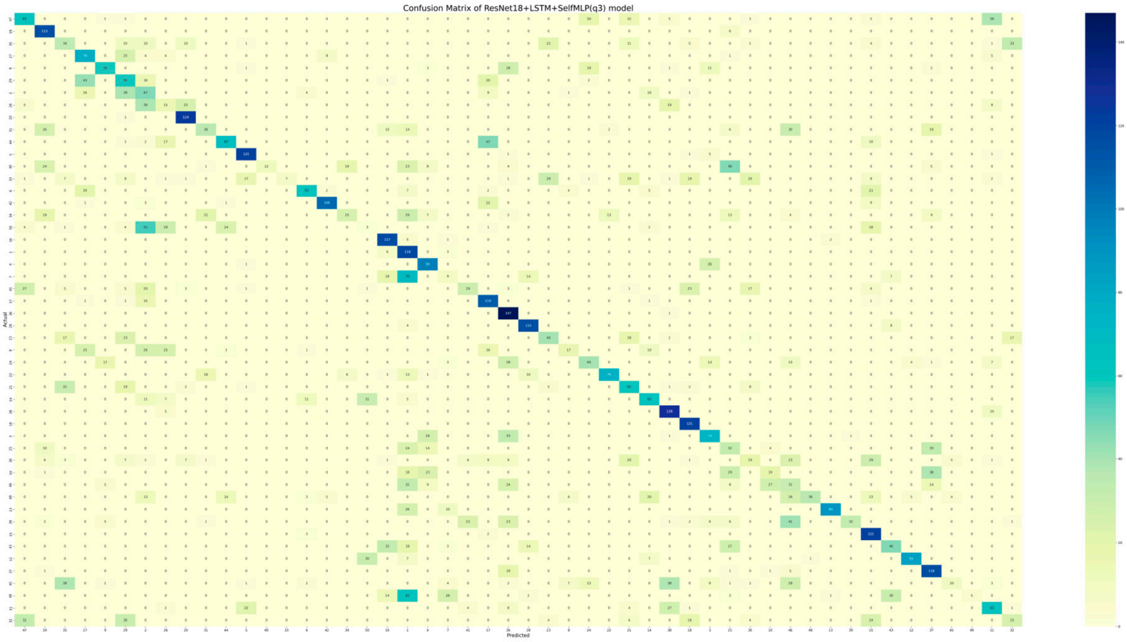

Figure S9: Confusion Matrix of ResNet18-LSTM-SelfMLP(q3) on Raw Dataset

ROC Curve: The ROC Curve of ResNet18-LSTM-SelfMLP(q3) on Raw Dataset is given below:

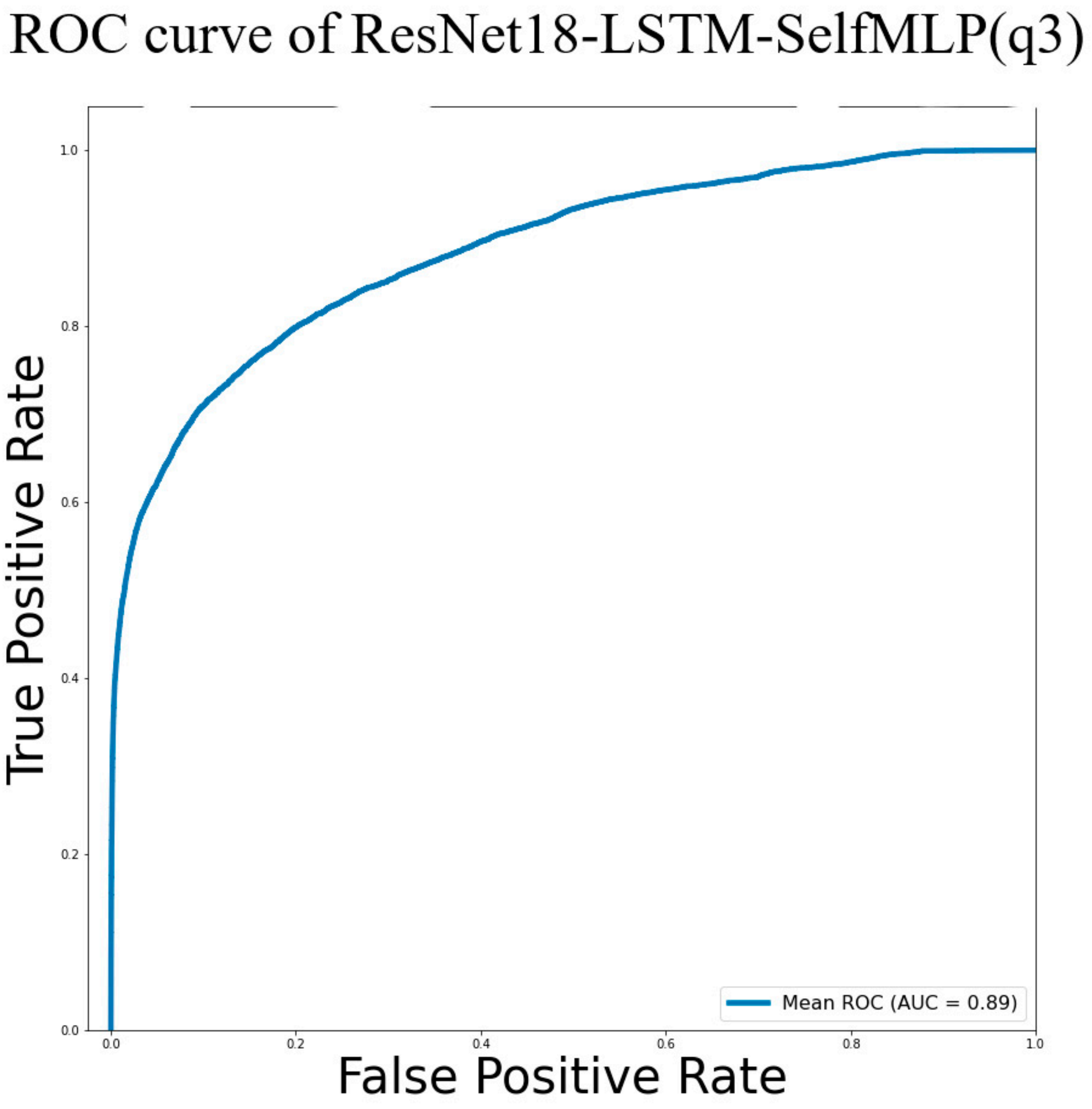

Figure S10: ROC Curve of ResNet18-LSTM-SelfMLP (q3) on Raw Dataset

Model: ResNet18-LSTM-SelfMLP(q5) on Raw Dataset

The accuracy and loss curves during the training of **ResNet18-LSTM-SelfMLP(q5)** on Raw Dataset is given Table S6:

Table S6: Accuracy and Loss curves of ResNet18-LSTM-SelfMLP(q3) training on raw dataset

<table>
<tr><th>Fold</th><th>Accuracy</th><th>Loss</th></tr>
<tr><td>1</td><td>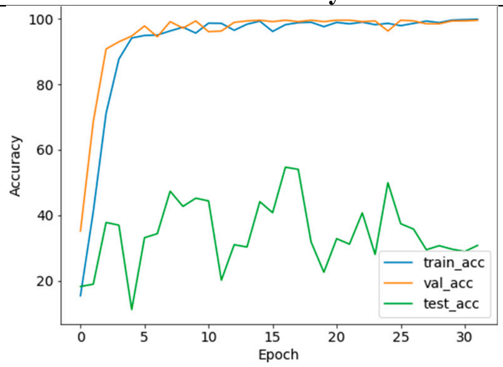
</td><td>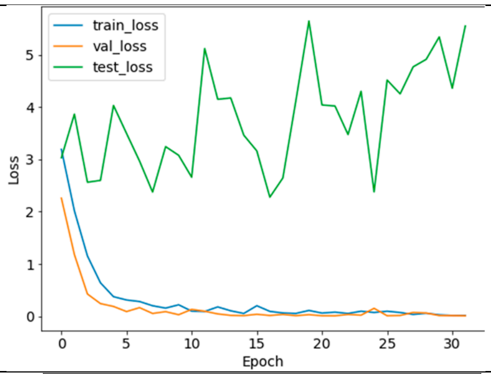
</td></tr>
<tr><td>2</td><td>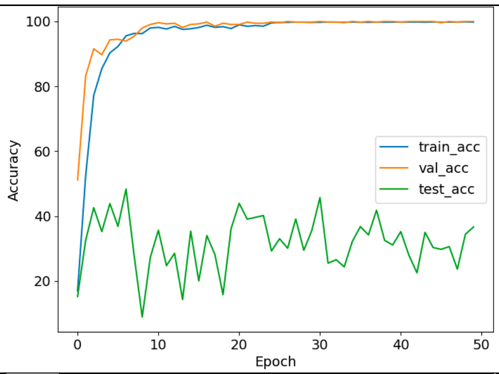
</td><td>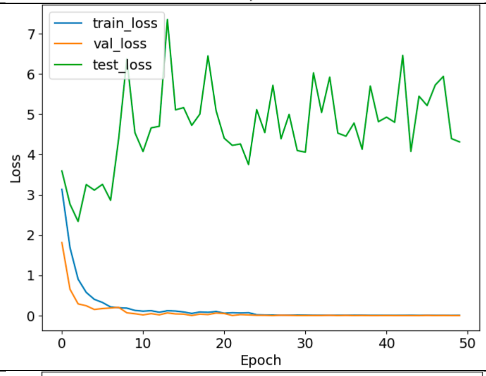
</td></tr>
<tr><td>3</td><td>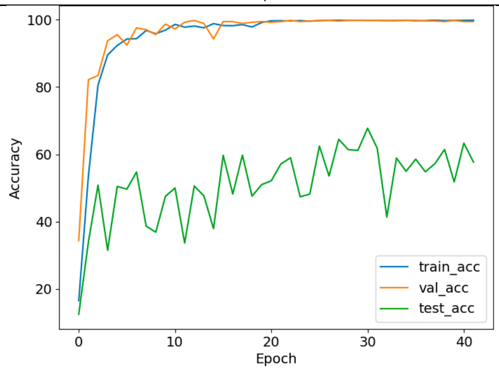
</td><td>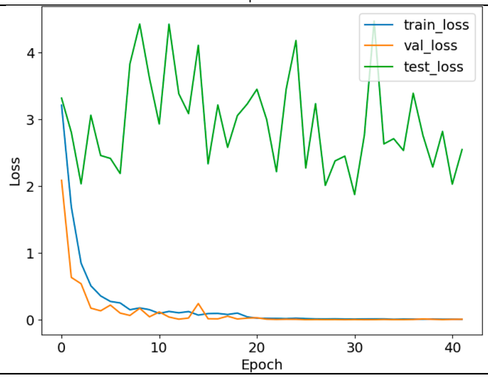
</td></tr>
</table>

4	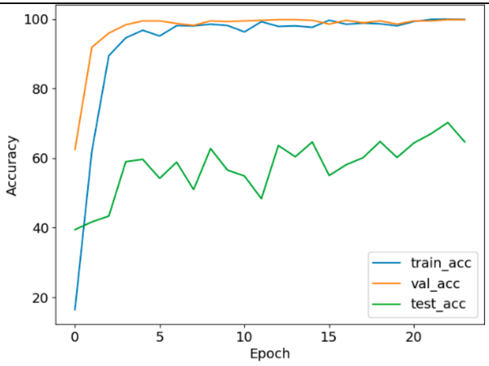Accuracy<br>Epoch<br>train_acc<br>val_acc<br>test_acc	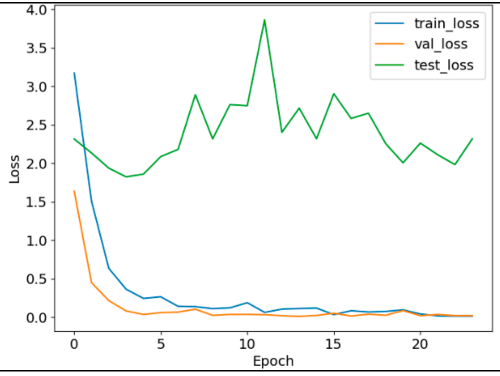Loss<br>Epoch<br>train_loss<br>val_loss<br>test_loss

Confusion Matrix: The Confusion Matrix of ResNet18-LSTM-SelfMLP(q5) on Raw Dataset is given below:

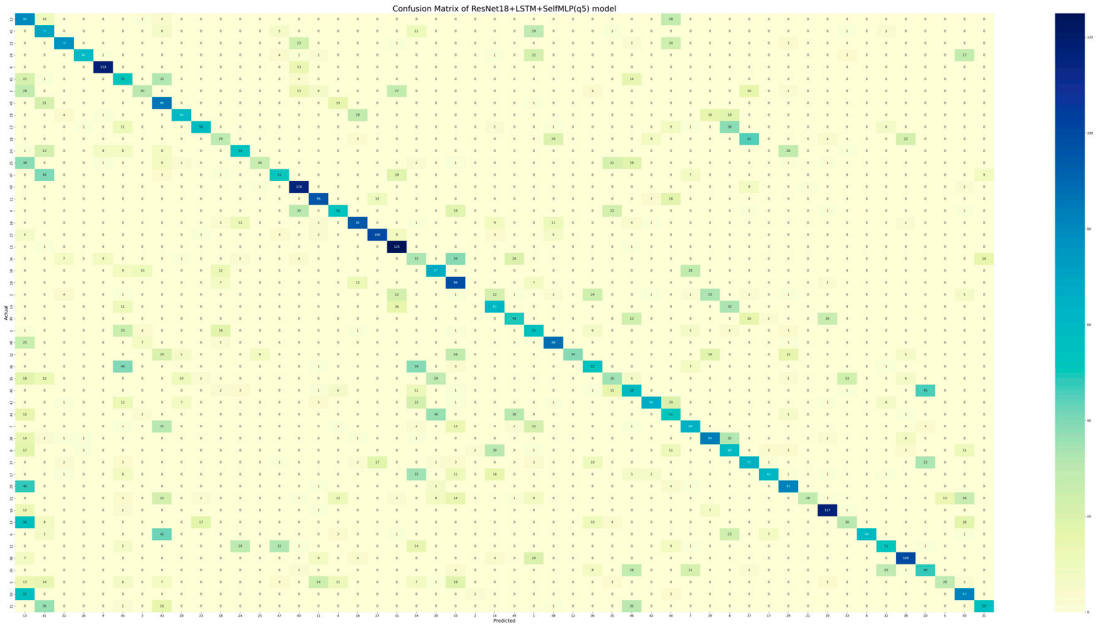

Figure S11: Confusion Matrix of ResNet18-LSTM-SelfMLP(q5) on Raw Dataset

ROC Curve: The ROC Curve of ResNet18-LSTM-SelfMLP+ LSTM + SelfMLP (q5) on Raw Dataset is given below:

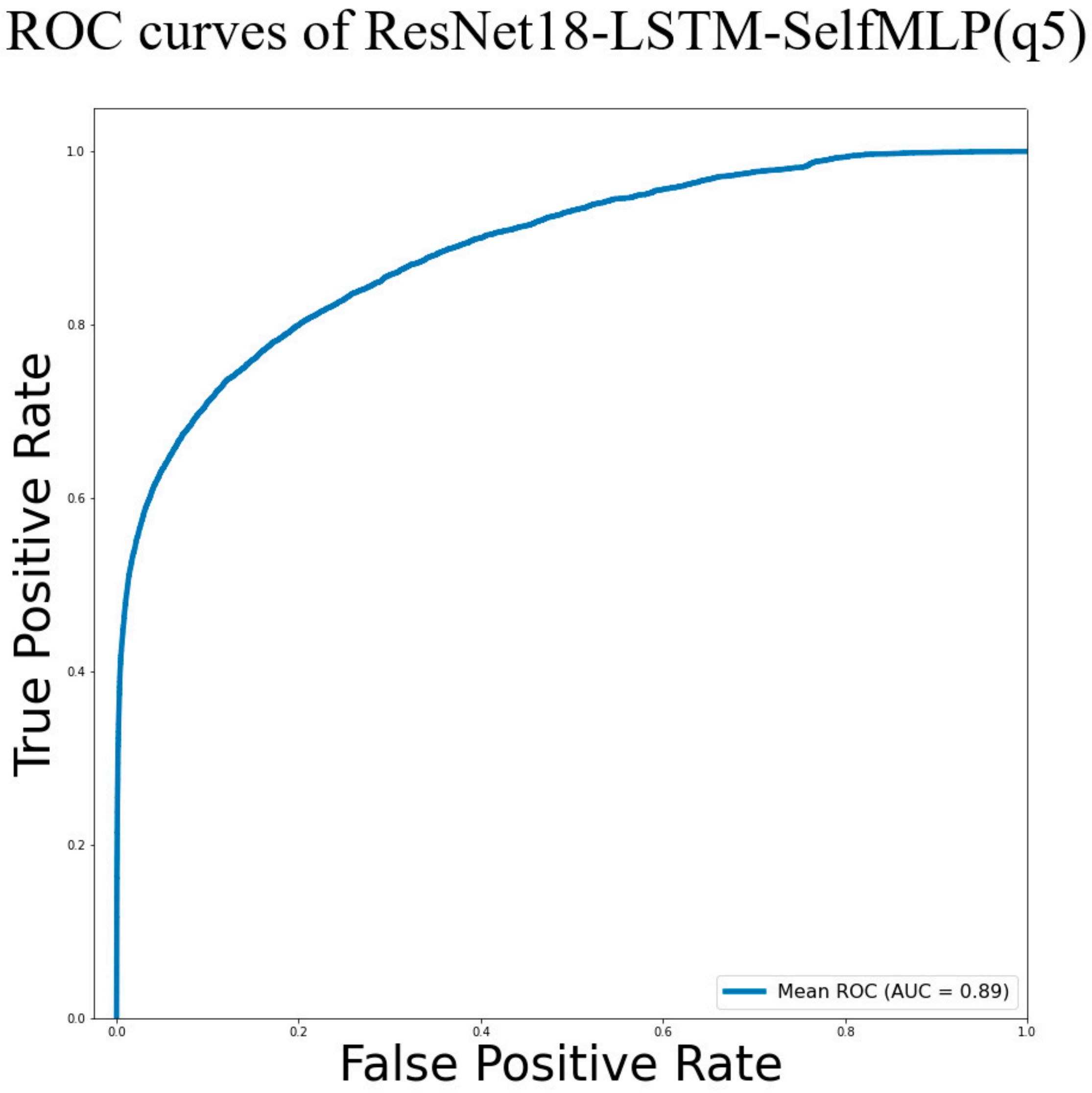

Figure S12: ROC Curve of ResNet18-LSTM-SelfMLP (q5) on Raw Dataset

Model: MobileNetV2-LSTM-SelfMLP (q1) on Segmented Dataset

The accuracy and loss curves during the training of **MobileNetV2-LSTM-SelfMLP (q1)** on Segmented Dataset is given Table S7:

Table S7: Accuracy and Loss curves of MobileNetV2-LSTM-SelfMLP (q1) training

Fold	Accuracy	Loss
1	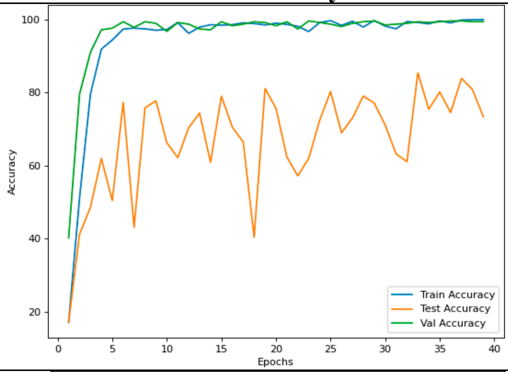	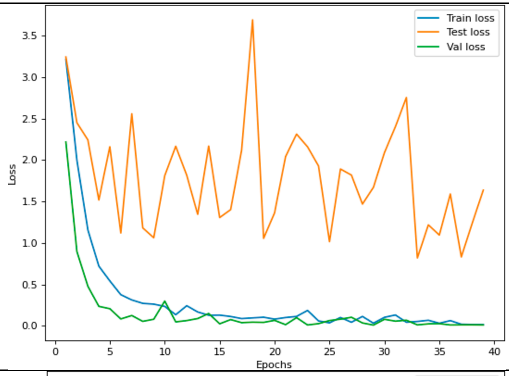
2	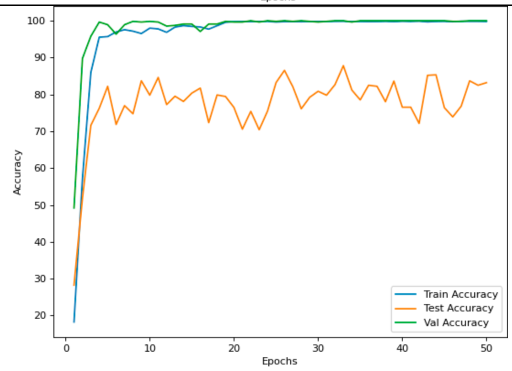	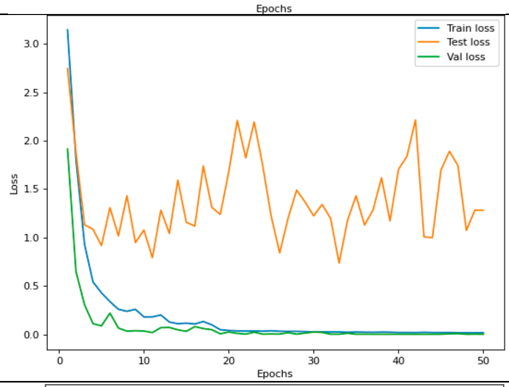
3	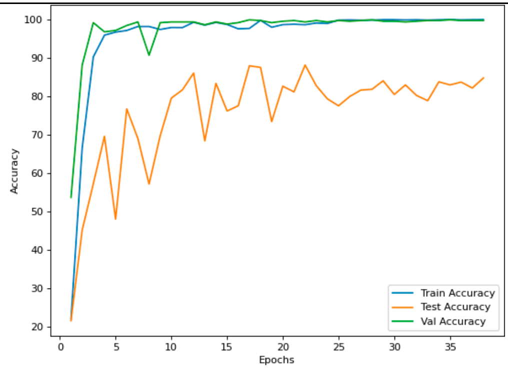	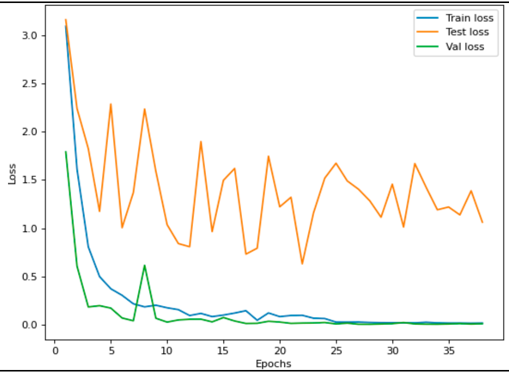

4	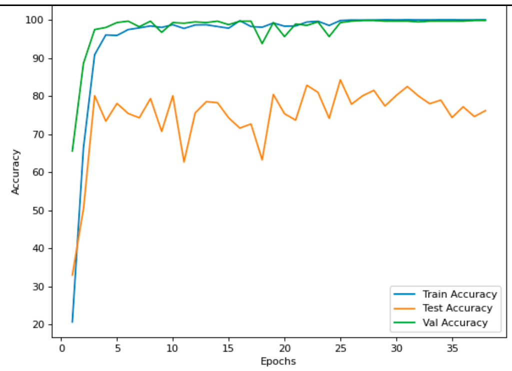Accuracy<br>Epochs<br>Train Accuracy<br>Test Accuracy<br>Val Accuracy	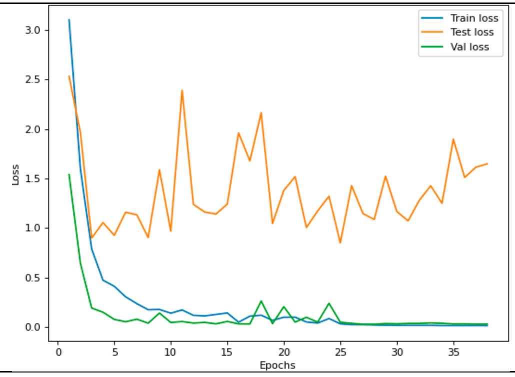Loss<br>Epochs<br>Train loss<br>Test loss<br>Val loss

Confusion Matrix: The Confusion Matrix of MobileNetV2-LSTM-SelfMLP (q1) on Segmented Dataset is given below:

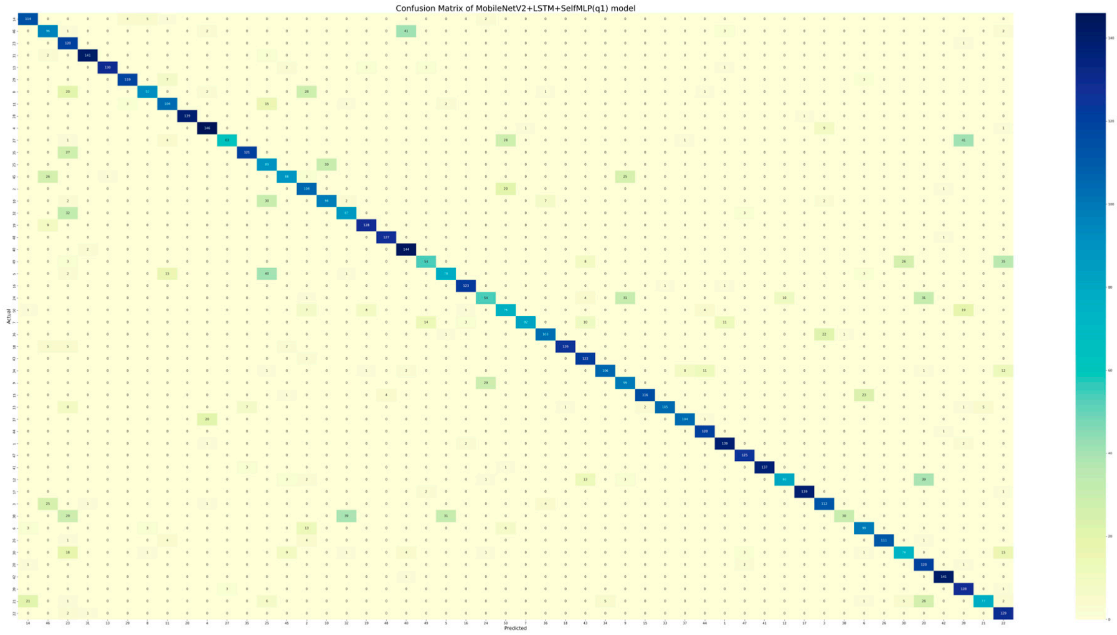

Figure S13: Confusion Matrix of MobileNetV2-LSTM-SelfMLP (q1) on Segmented Dataset

ROC Curve: The ROC Curve of MobileNetV2-LSTM-SelfMLP (q1) on Segmented Dataset is given below:

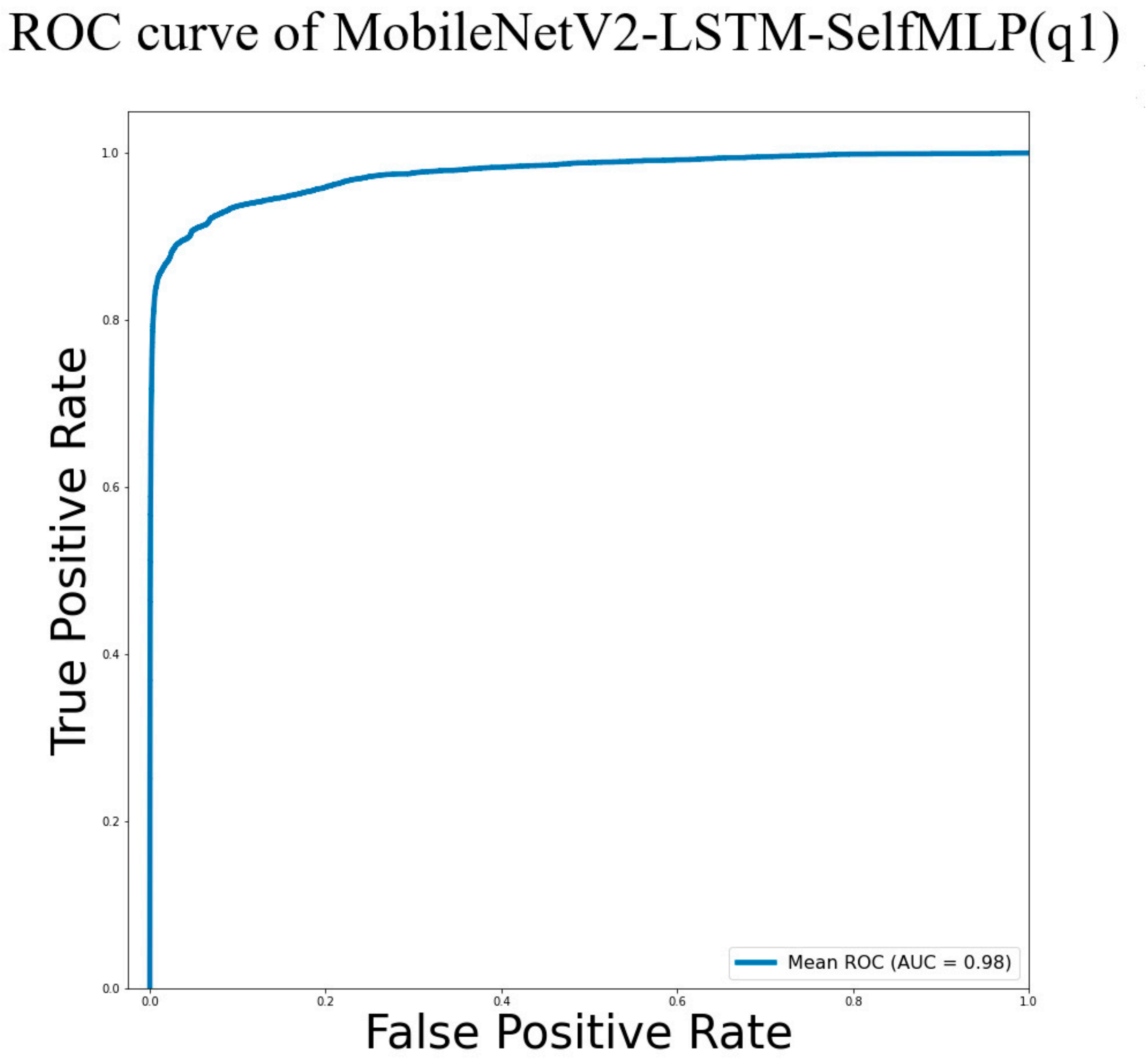

Figure S14: ROC Curve of MobileNetV2-LSTM-SelfMLP (q1) on Segmented Dataset

Model: MobileNetV2-LSTM-SelfMLP (q3) on Segmented Dataset

The accuracy and loss curves during the training of **MobileNetV2-LSTM-SelfMLP (q3)** on Segmented Dataset is given Table S8:

Table S8: Accuracy and Loss curves of MobileNetV2-LSTM-SelfMLP (q3) training

Fold	Accuracy	Loss
1	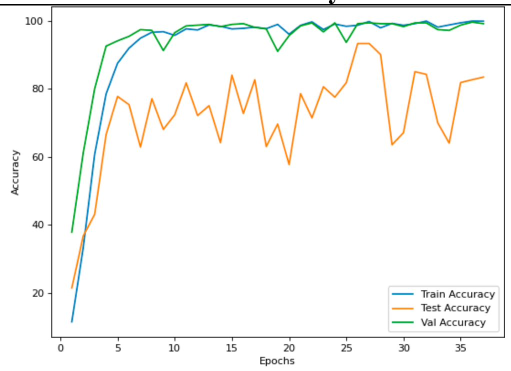	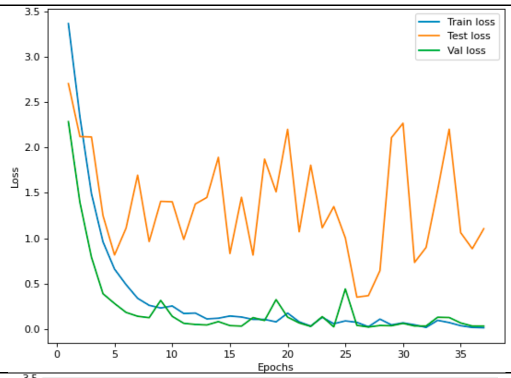
2	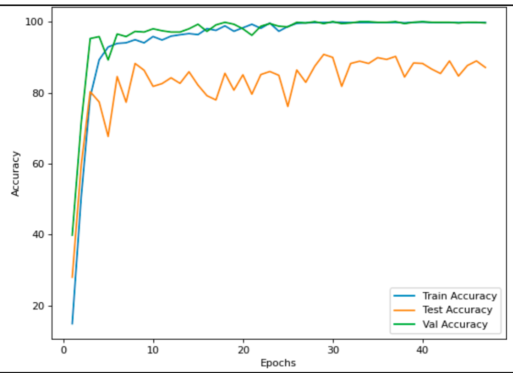	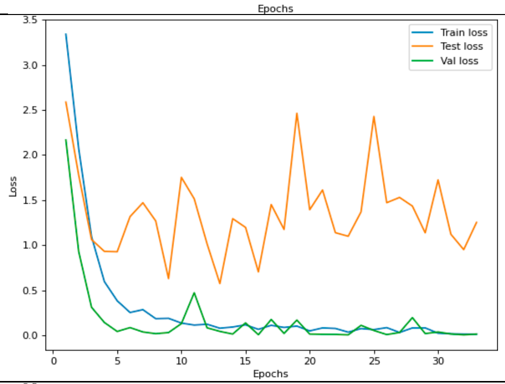
3	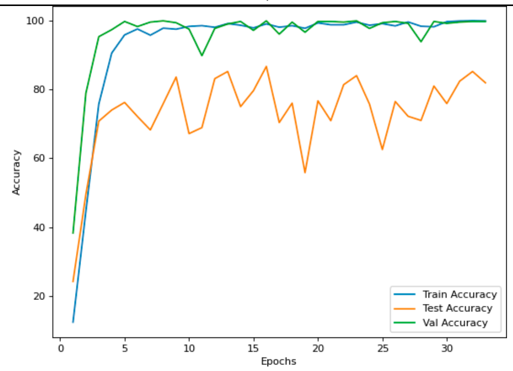	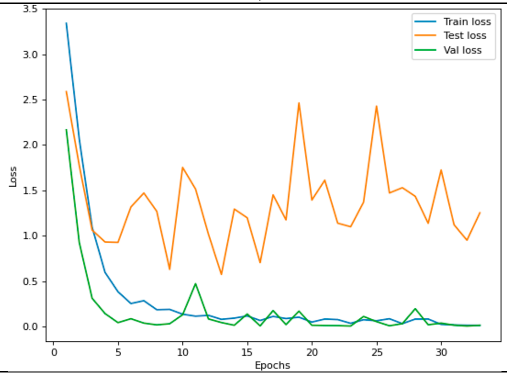

4

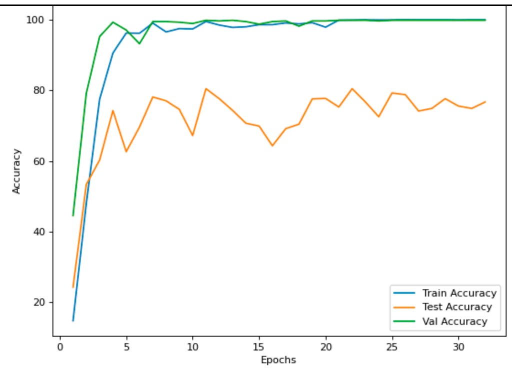
Accuracy
Epochs
Train Accuracy
Test Accuracy
Val Accuracy

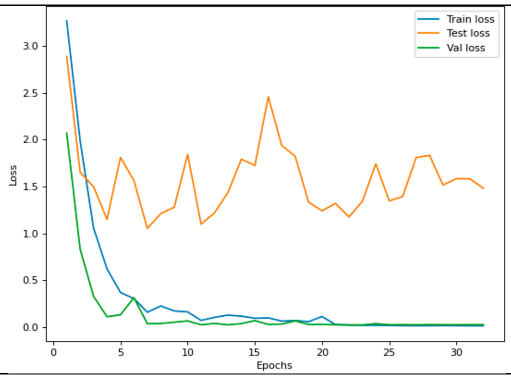
Loss
Epochs
Train loss
Test loss
Val loss

Confusion Matrix: The Confusion Matrix of MobileNetV2-LSTM-SelfMLP (q3) on Segmented Dataset is given below:

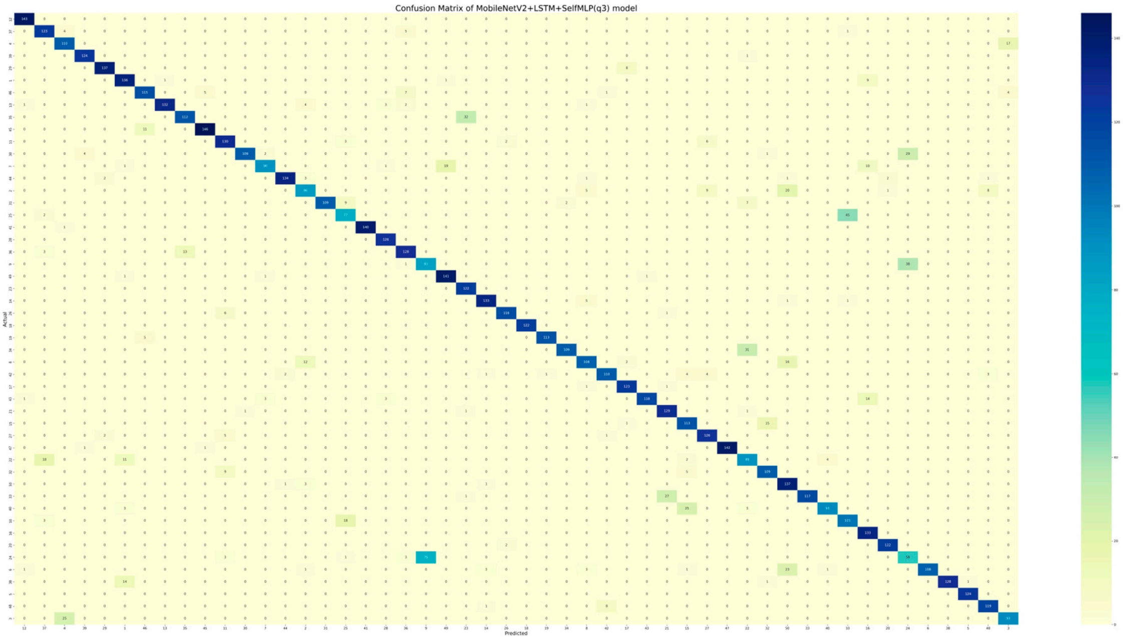

Figure S15: Confusion Matrix of MobileNetV2-LSTM-SelfMLP (q3) on Segmented Dataset

ROC Curve: The ROC Curve of MobileNetV2-LSTM-SelfMLP (q3) on Segmented Dataset is given below:

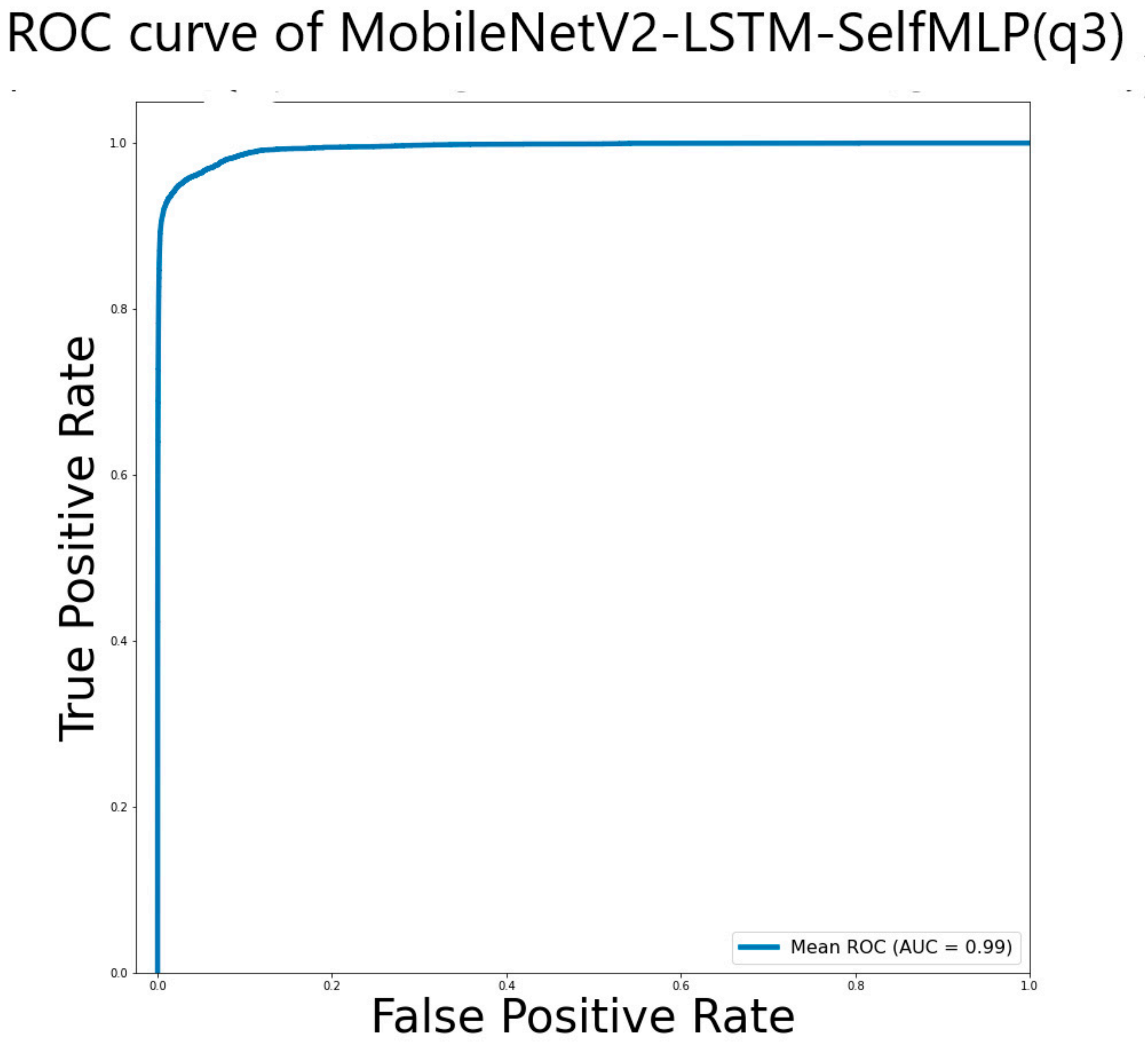

Figure S16: ROC Curve of MobileNetV2-LSTM-SelfMLP (q3) on Segmented Dataset

Model: MobileNetV2-LSTM-SelfMLP (q5) on Segmented Dataset

The accuracy and loss curves during the training of **MobileNetV2-LSTM-SelfMLP (q5)** on Segmented Dataset is given Table S9:

Table S9: Accuracy and Loss curves of MobileNetV2-LSTM-SelfMLP (q5) training

<table>
<tr><th>Fold</th><th>Accuracy</th><th>Loss</th></tr>
<tr><td>1</td><td>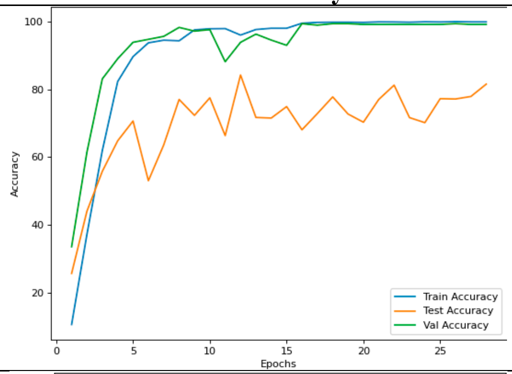
</td><td>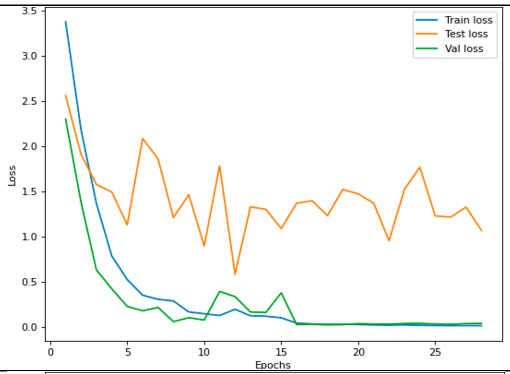
</td></tr>
<tr><td>2</td><td>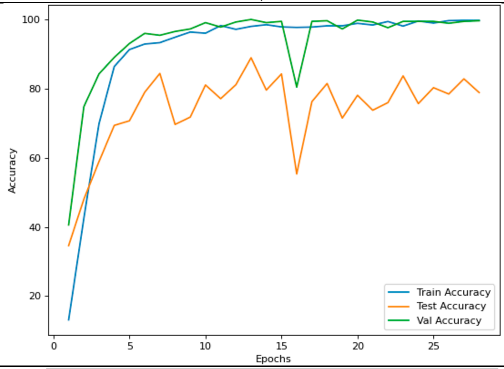
</td><td>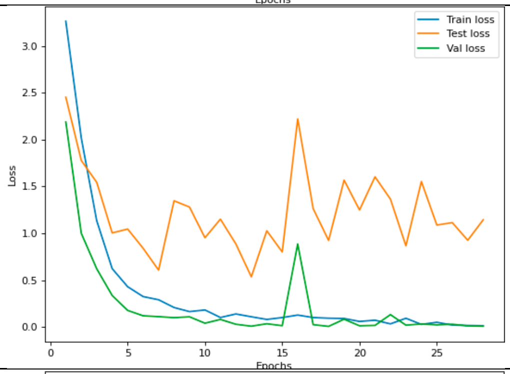
</td></tr>
<tr><td>3</td><td>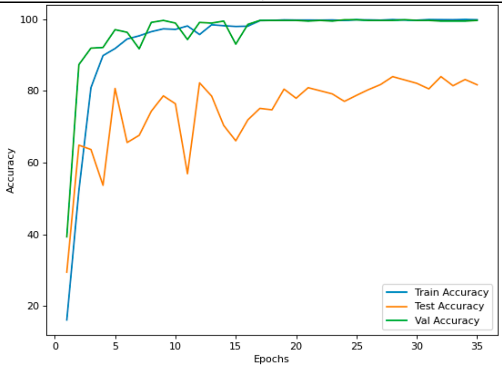
</td><td>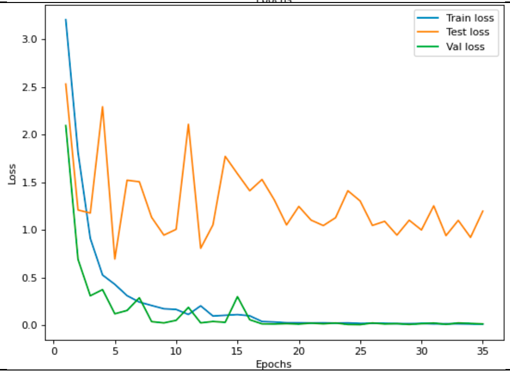
</td></tr>
</table>

4	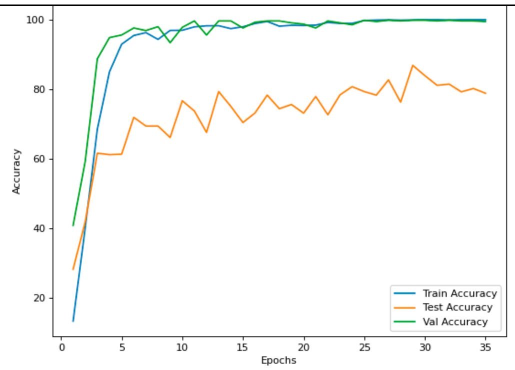 Accuracy<br>Epochs<br>Train Accuracy<br>Test Accuracy<br>Val Accuracy	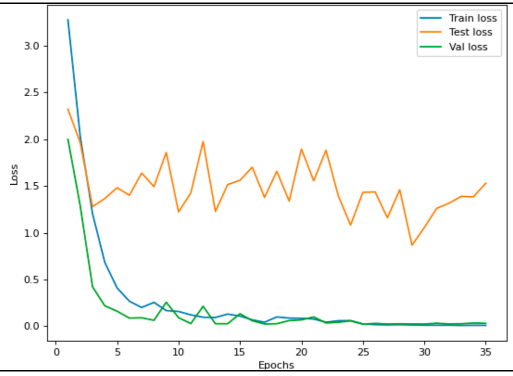 Loss<br>Epochs<br>Train loss<br>Test loss<br>Val loss

Confusion Matrix: The Confusion Matrix of MobileNetV2-LSTM-SelfMLP (q5) on Segmented Dataset is given below:

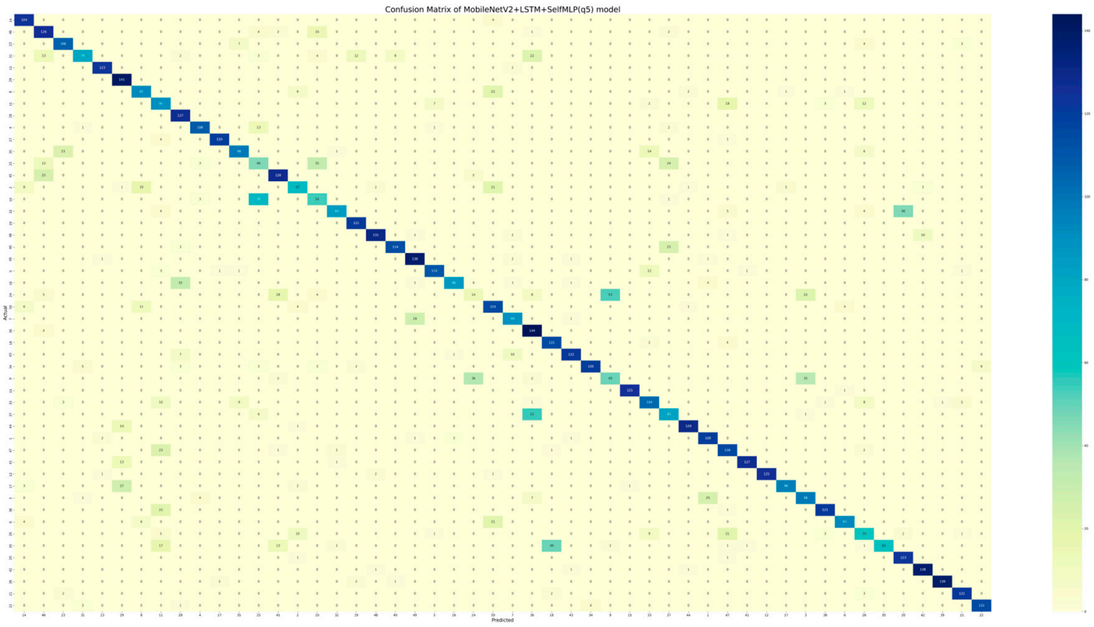

Figure S17: Confusion Matrix of MobileNetV2-LSTM-SelfMLP (q5) on Segmented Dataset

ROC Curve: The ROC Curve of MobileNetV2-LSTM-SelfMLP (q5) on Segmented Dataset is given below:

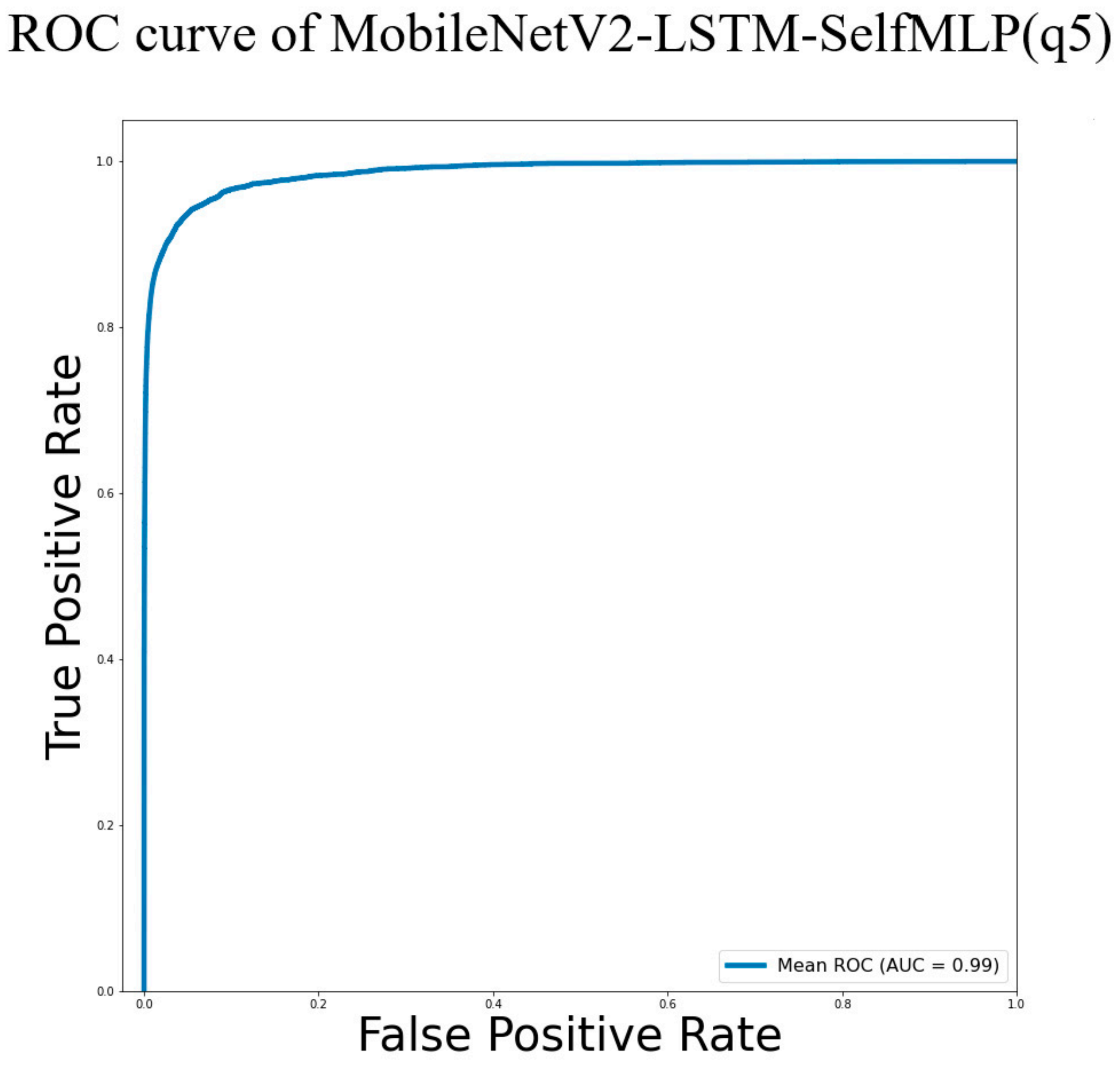

Figure S18: ROC Curve of MobileNetV2-LSTM-SelfMLP (q5) on Segmented Dataset

Model: ResNet18-LSTM-SelfMLP(q1) on Segmented Dataset

The accuracy and loss curves during the training of **ResNet18-LSTM-SelfMLP(q1)** on Segmented Dataset is given Table S10:

Table S10: Accuracy and Loss curves of ResNet18-LSTM-SelfMLP(q1) training

Fold	Accuracy	Loss
1	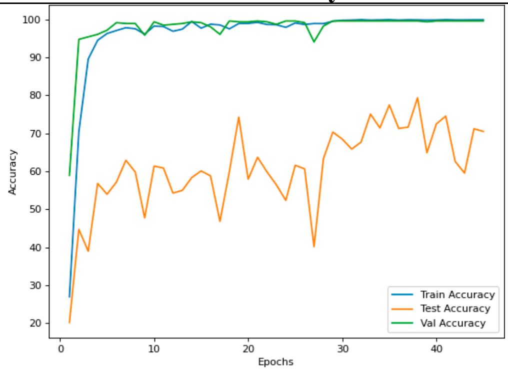	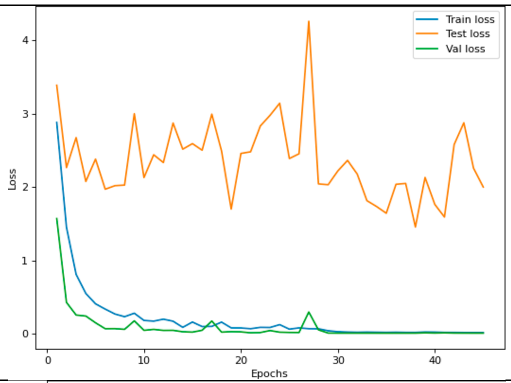
2	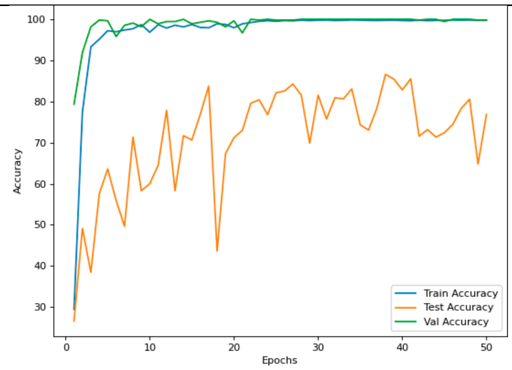	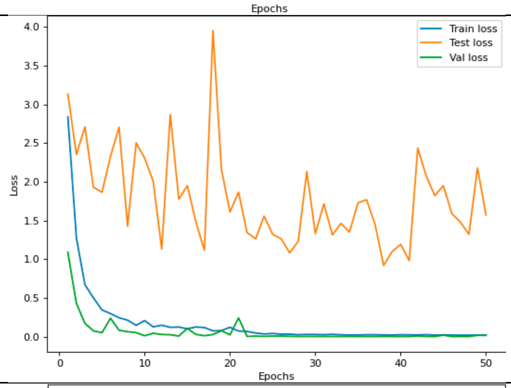
3	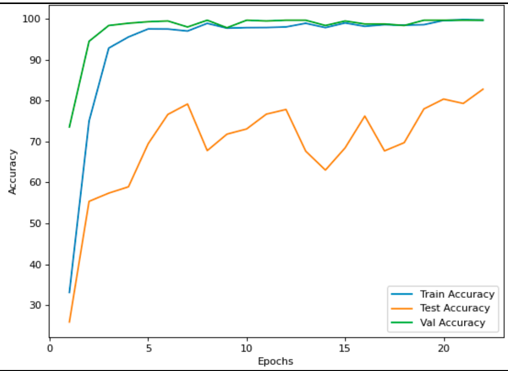	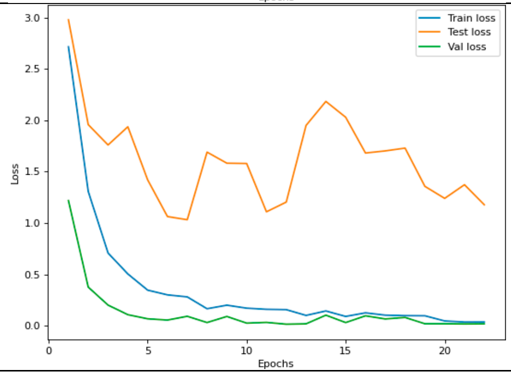

4	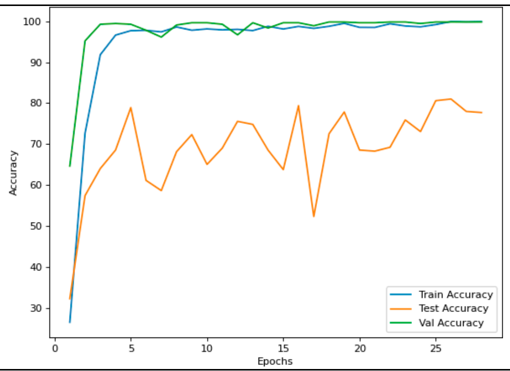	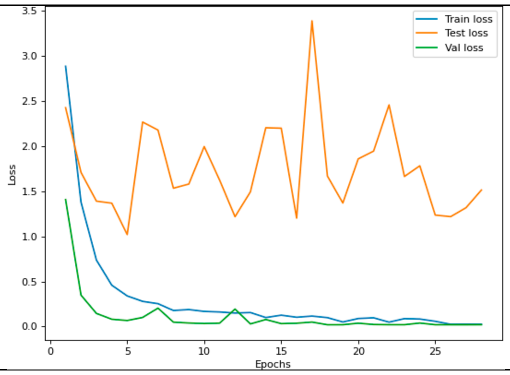

Accuracy
Epochs
Train Accuracy
Test Accuracy
Val Accuracy

Loss
Epochs
Train loss
Test loss
Val loss

Confusion Matrix: The Confusion Matrix of ResNet18-LSTM-SelfMLP(q1) on Segmented Dataset is given below:

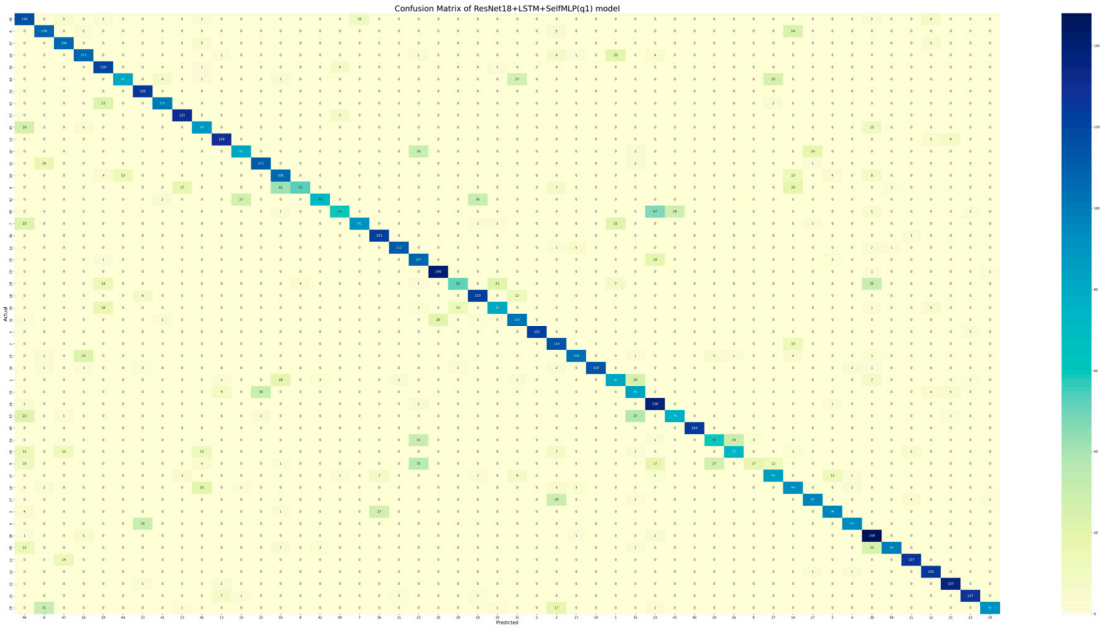

Figure S19: Confusion Matrix of ResNet18-LSTM-SelfMLP(q1) on Segmented Dataset

ROC Curve: The ROC Curve of ResNet18-LSTM-SelfMLP(q1) on Segmented Dataset is given below:

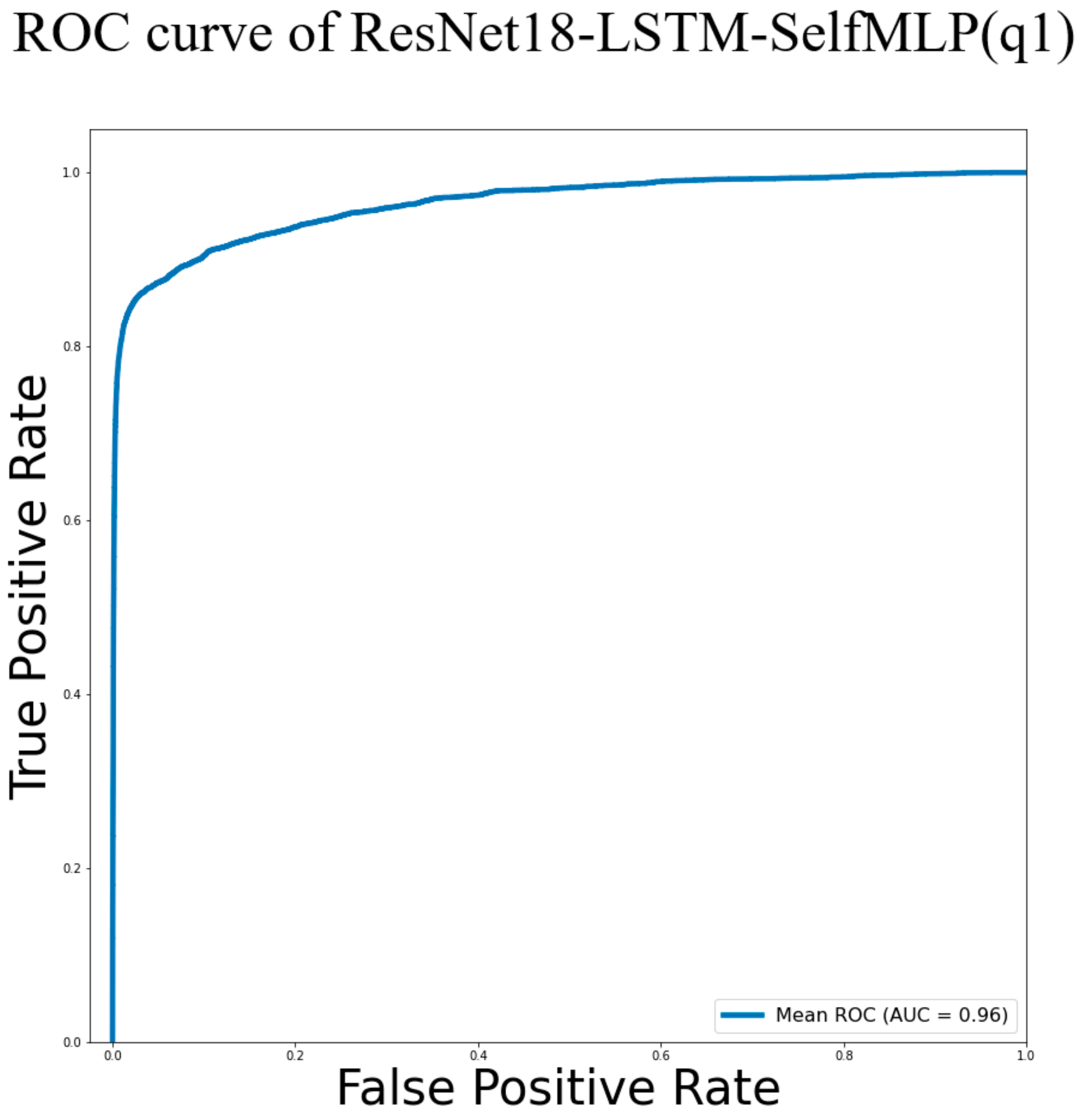

Figure S20: ROC Curve of ResNet18-LSTM-SelfMLP(q1) on Segmented Dataset

Model: ResNet18-LSTM-SelfMLP(q3) on Segmented Dataset

The accuracy and loss curves during the training of **ResNet18-LSTM-SelfMLP(q3)** on Segmented Dataset is given Table S11:

Table S11: Accuracy and Loss curves of ResNet18-LSTM-SelfMLP(q3) training

**Fold**	**Accuracy**	**Loss**
1	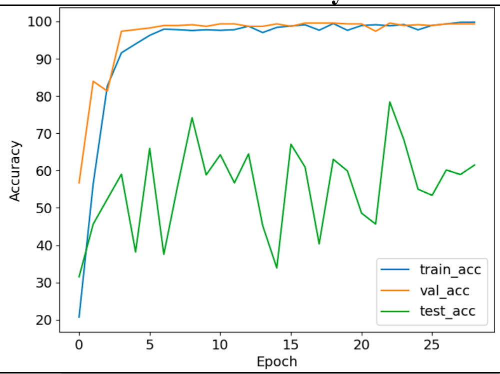	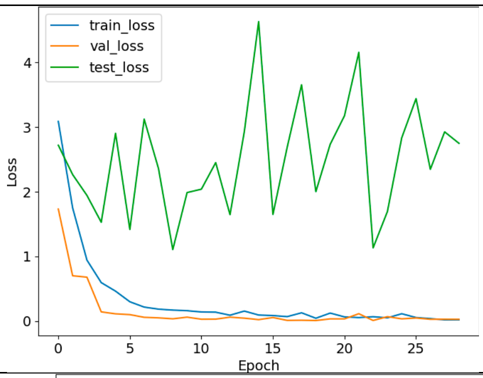
2	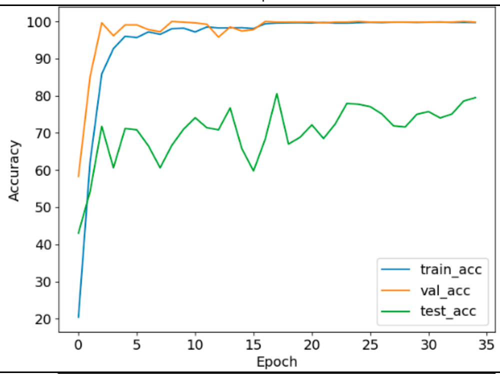	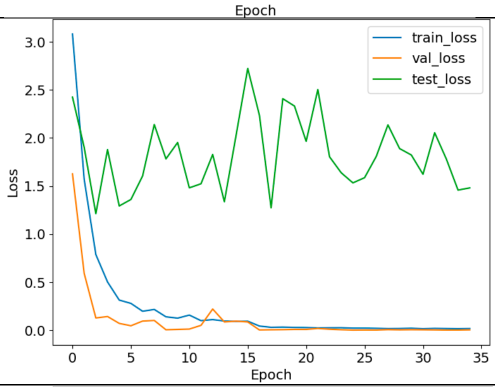
3	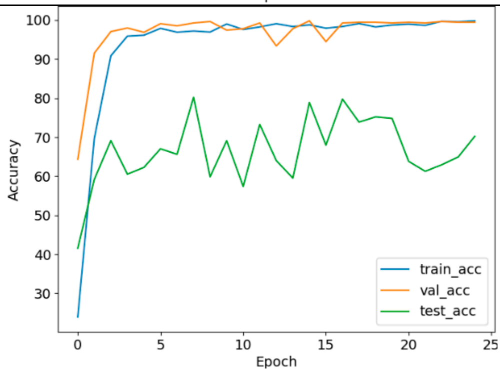	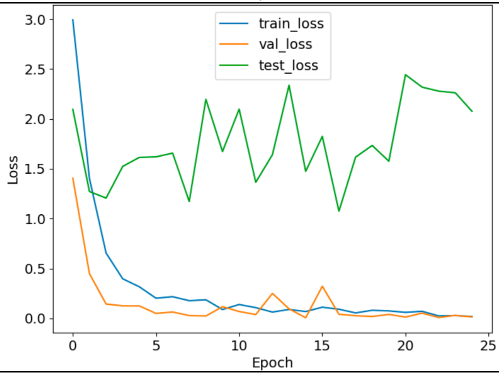

4	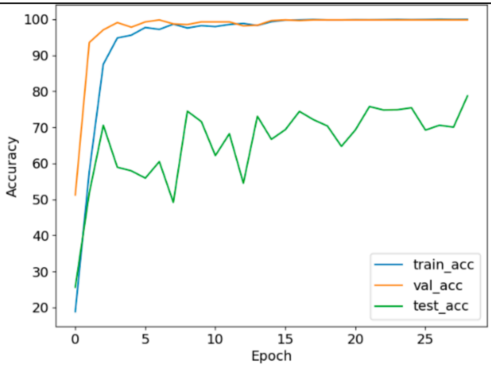Accuracy<br>Epoch<br>train_acc<br>val_acc<br>test_acc	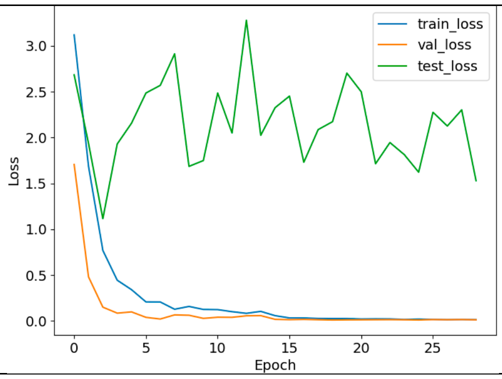Loss<br>Epoch<br>train_loss<br>val_loss<br>test_loss

Confusion Matrix: The Confusion Matrix of ResNet18-LSTM-SelfMLP(q3) on Segmented Dataset is given below:

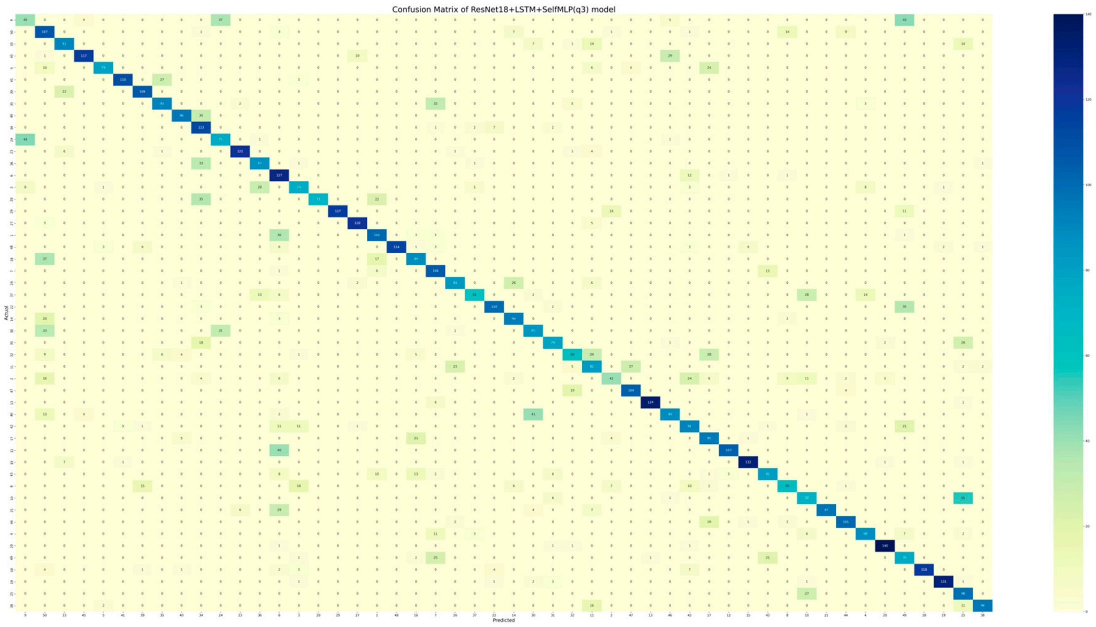

Figure S21: Confusion Matrix of ResNet18-LSTM-SelfMLP(q3) on Segmented Dataset

ROC Curve: The ROC Curve of ResNet18-LSTM-SelfMLP(q3) on Segmented Dataset is given below:

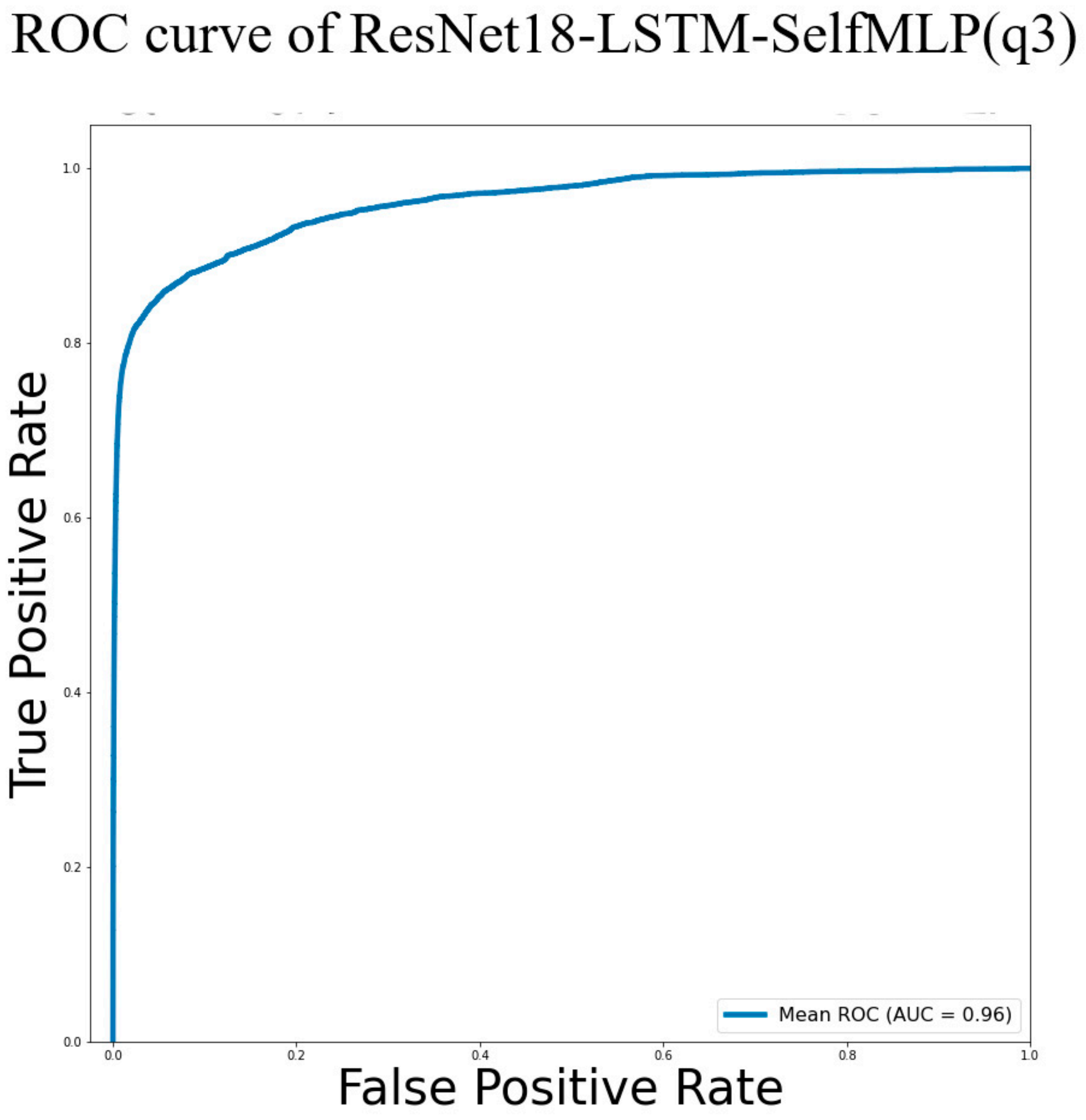

Figure S22: ROC Curve of ResNet18-LSTM-SelfMLP(q3) on Segmented Dataset

Model: ResNet18-LSTM-SelfMLP(q5) on Segmented Dataset

The accuracy and loss curves during the training of **ResNet18-LSTM-SelfMLP(q5)** on Segmented Dataset is given Table S12:

Table S22: Accuracy and Loss curves of ResNet18-LSTM-SelfMLP(q5) training

<table>
<tr><th>Fold</th><th>Accuracy</th><th>Loss</th></tr>
<tr><td>1</td><td>
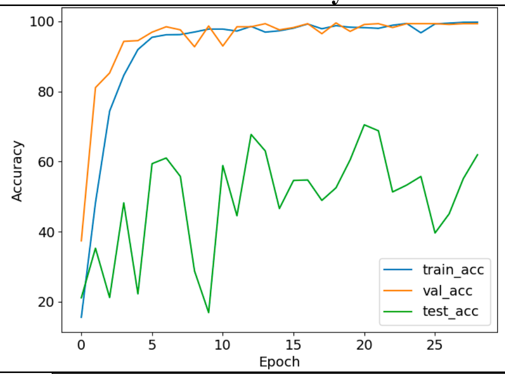

</td><td>
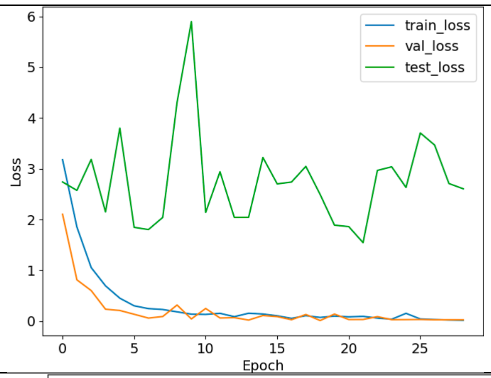

</td></tr>
<tr><td>2</td><td>
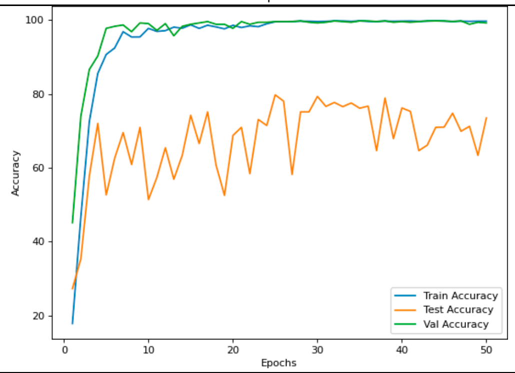

</td><td>
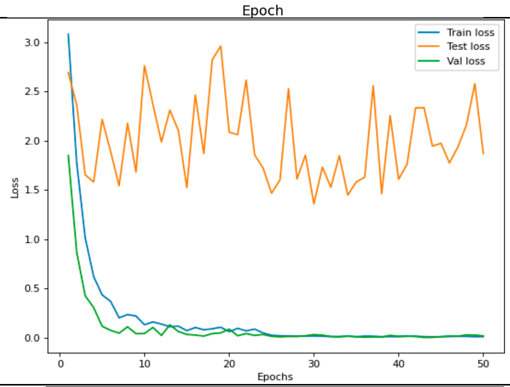

</td></tr>
<tr><td>3</td><td>
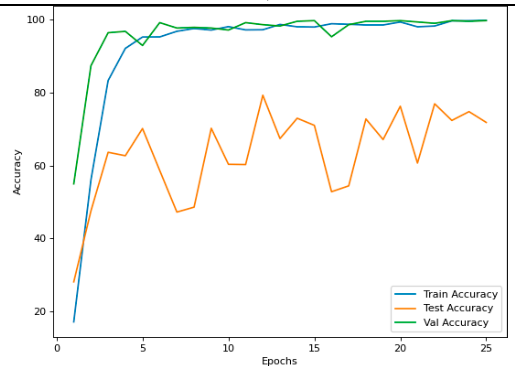

</td><td>
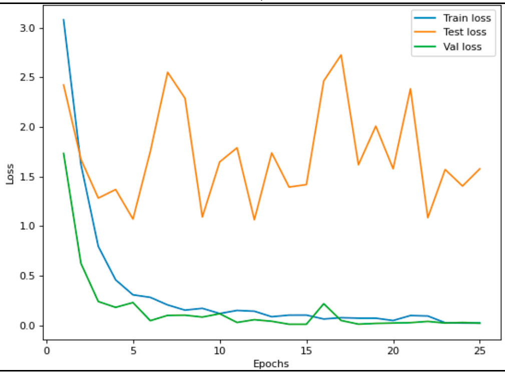

</td></tr>
</table>

4	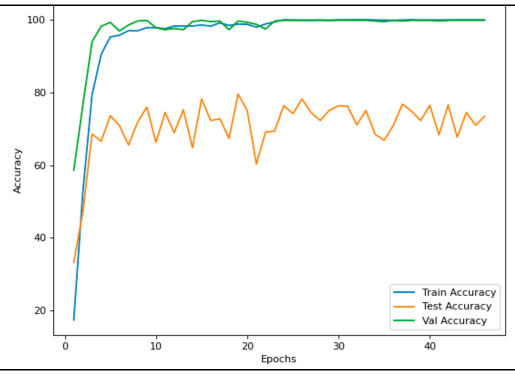 Accuracy<br>Epochs<br>Train Accuracy<br>Test Accuracy<br>Val Accuracy	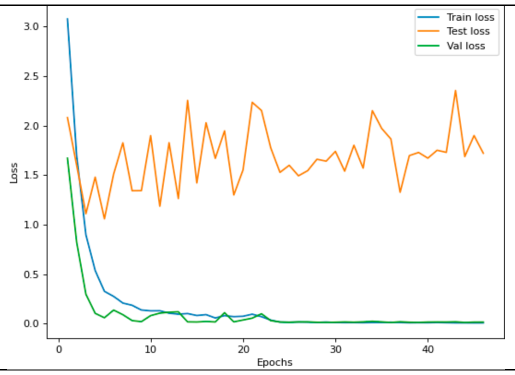 Loss<br>Epochs<br>Train loss<br>Test loss<br>Val loss

Confusion Matrix: The Confusion Matrix of ResNet18-LSTM-SelfMLP(q5) on Segmented Dataset is given below:

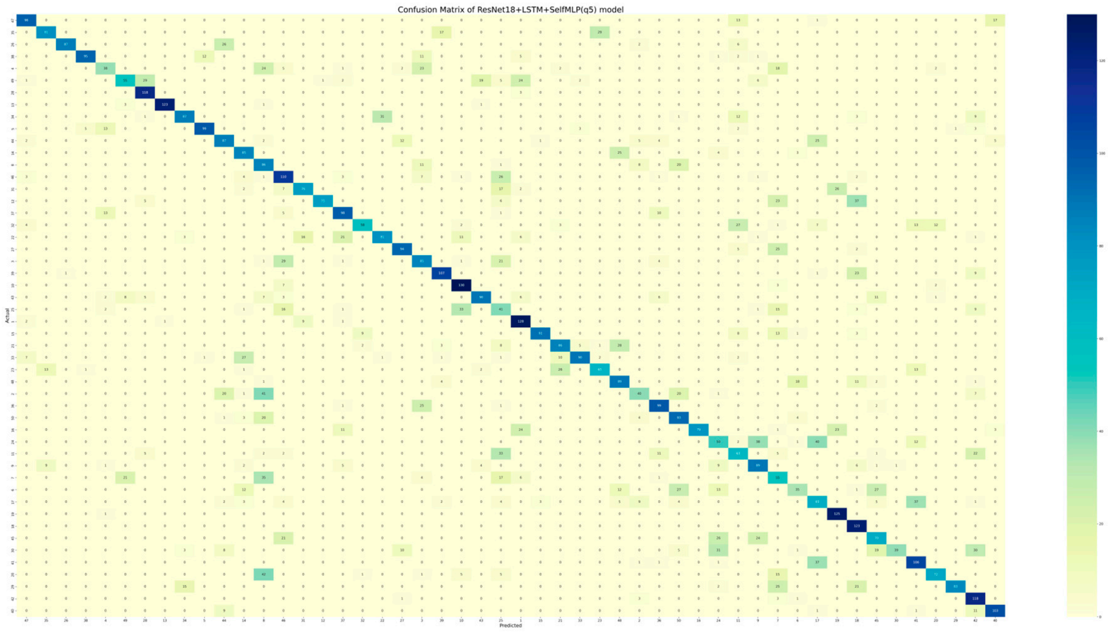

Figure S23: Confusion Matrix of ResNet18-LSTM-SelfMLP(q5) on Segmented Dataset

ROC Curve: The ROC Curve of ResNet18-LSTM-SelfMLP(q5) on Segmented Dataset is given below:

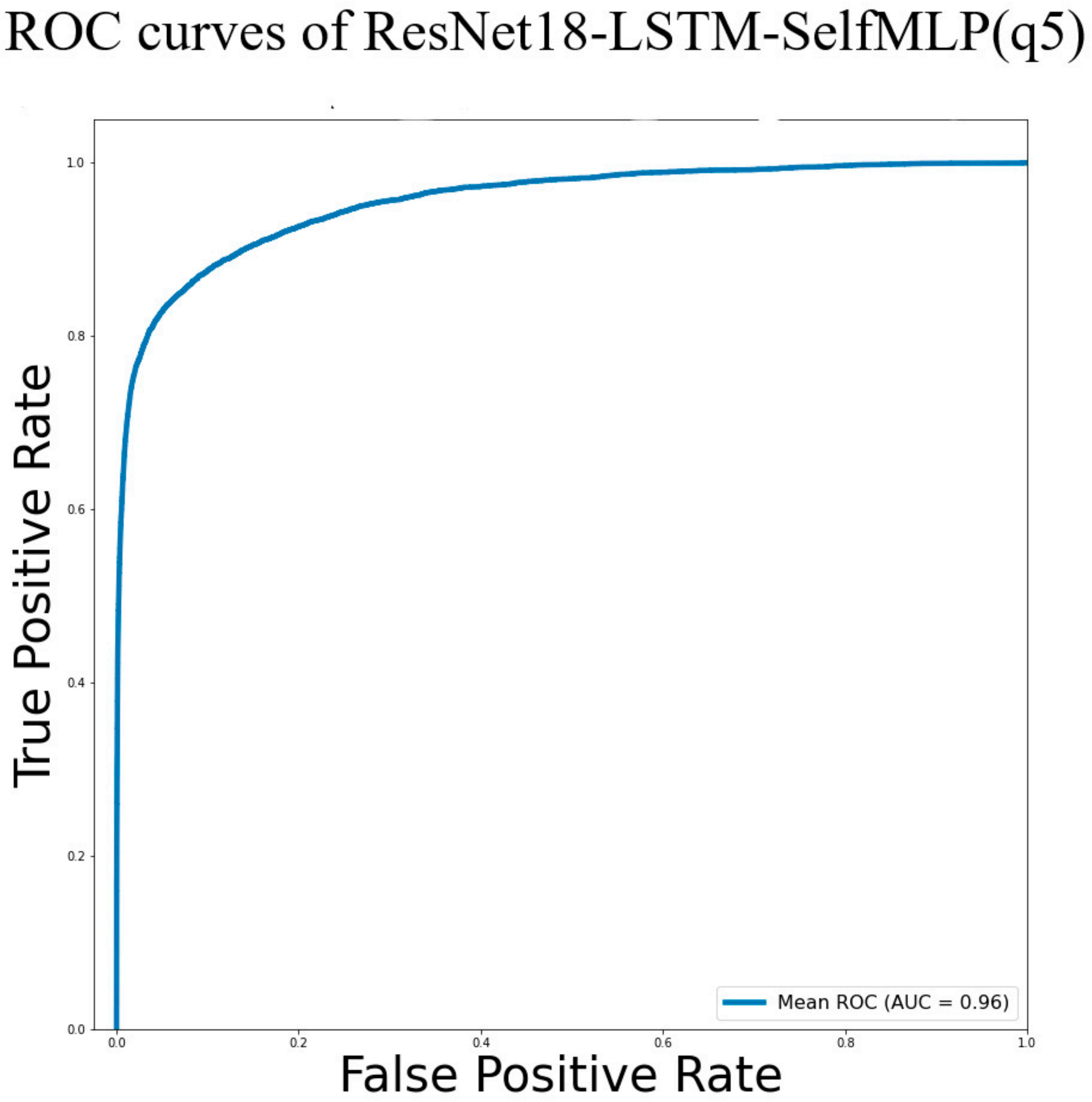

Figure S24: ROC Curve of ResNet18-LSTM-SelfMLP(q5) on Segmented Dataset
